# Quantitative Insights into the Fast Pyrolysis of Extracted Cellulose, Hemicelluloses, and Lignin

**DOI:** 10.1002/cssc.201700984

**Published:** 2017-07-25

**Authors:** Marion Carrier, Michael Windt, Bernhard Ziegler, Jörn Appelt, Bodo Saake, Dietrich Meier, Anthony Bridgwater

**Affiliations:** ^1^ European Bioenergy Research Institute Aston University Birmingham B4 7ET UK; ^2^ Thünen Institute of Wood Research Bio-based Resources and Materials Leuschnerstr. 91 21031 Hamburg Germany; ^3^ University of Hamburg Chemical Wood Technology Leuschnerstr 91 21031 Hamburg Germany

**Keywords:** biomass, isotopic labeling, NMR spectroscopy, polymers, reaction mechanisms

## Abstract

The transformation of lignocellulosic biomass into bio‐based commodity chemicals is technically possible. Among thermochemical processes, fast pyrolysis, a relatively mature technology that has now reached a commercial level, produces a high yield of an organic‐rich liquid stream. Despite recent efforts to elucidate the degradation paths of biomass during pyrolysis, the selectivity and recovery rates of bio‐compounds remain low. In an attempt to clarify the general degradation scheme of biomass fast pyrolysis and provide a quantitative insight, the use of fast pyrolysis microreactors is combined with spectroscopic techniques (i.e., mass spectrometry and NMR spectroscopy) and mixtures of unlabeled and ^13^C‐enriched materials. The first stage of the work aimed to select the type of reactor to use to ensure control of the pyrolysis regime. A comparison of the chemical fragmentation patterns of “primary” fast pyrolysis volatiles detected by using GC‐MS between two small‐scale microreactors showed the inevitable occurrence of secondary reactions. In the second stage, liquid fractions that are also made of primary fast pyrolysis condensates were analyzed by using quantitative liquid‐state ^13^C NMR spectroscopy to provide a quantitative distribution of functional groups. The compilation of these results into a map that displays the distribution of functional groups according to the individual and main constituents of biomass (i.e., hemicelluloses, cellulose and lignin) confirmed the origin of individual chemicals within the fast pyrolysis liquids.

## Introduction

The fast pyrolysis of plant biomass has now reached the technological and commercial maturity to convert solid materials into bio‐oil.^1^ Expected formerly to provide a solution for the replacement of fossil‐based liquid products, these bio‐oils have been seen more recently as potential feedstocks for chemicals from an integrated biorefinery perspective.^2^ However, a number of concerns with regard to the quality of fast pyrolysis bio‐oil have been raised (i.e., instability, high variability in chemical composition, high water content, immiscibility with petroleum‐derived fuels, changing viscosity, phase separation) that prevent its upgrading for commercial applications.^3^ In particular, the high level of oxygen in fast pyrolysis oils requires the application of intensive post‐treatments to deoxygenate these liquids selectively, which has resulted in intense scientific activity in the field of catalytic fast pyrolysis^4^ and bio‐oil upgrading in the last decade.^5^ A considerable number of catalysts has been developed as a result of the chemical diversity of the components in bio‐oils.^4^ Whatever the processing approach (i.e., ex situ or in situ) or catalysis approach (i.e., homogeneous or heterogeneous) used, it is reasonable to think that a better understanding of the origin of the constituent chemicals in bio‐oil could have a beneficial impact on the overall performance of these processes.

The understanding of biomass pyrolysis mechanisms creates a real challenge if we consider the large diversity of the types of biomass and fast pyrolysis technologies. For several decades, researchers have tried to elucidate the main degradation pathways for biomass fast pyrolysis modelling.^6^, ^7^, ^8^, ^9^, ^10^, ^11^ As a result, the overall degradation scheme of biomass fast pyrolysis is seen as an interplay between physical and chemical events, which are often impossible to separate.^12^ Evans and Milne described a degradation scheme that indicates the main degradation pathways according to process conditions with byproducts classified as primary, secondary and tertiary.^9^ Recently, researchers have questioned the nature of the proposed mechanisms. Indeed, the chemical aspect of biomass fast pyrolysis can be described as a combination of parallel and successive reactions of a nonionic and ionic nature.^13^ If radical mechanisms are invoked and are predominant in coal pyrolysis,^14^ recent experimental evidence^6^, ^7^, ^15^ and theoretical calculations^16^, ^17^ for biomass fast pyrolysis suggest the predominance of nonionic reactions during the primary pyrolysis stage. This ongoing discussion on the importance and predominance of the ionic and/or nonionic character of fast pyrolysis reactions^13^ has provided important clues that have not yet been used to rationalize the degradation modes. This is mainly because of a lack of rigorous analytical methodology and the absence of the control of reaction regimes that lead to contradictory interpretations of mechanisms. For example, the control of the heating rate is of importance if we discuss types of mechanisms as the heating rates selected have a direct influence on the chemical composition of the bio‐oil. Experimental evidence has shown a significant change in the quality of bio‐oil if we use slow or fast pyrolysis.^18^

The chemical composition of bio‐oil is process‐dependent and affected by the nature of the lignocellulose feed material. In an attempt to identify and delineate the chemical reactions related to the transformation of individual biopolymers (i.e., cellulose, hemicelluloses and lignin), researchers have opted for the use of model compounds and different analytical strategies. Most of the degradation pathways that have been suggested to date are based on the thermal degradation of model compounds, which leads to oversimplified degradation schemes and a biased picture of the composition of bio‐oil. However, these studies have been instrumental to reveal key patterns. Indeed, several pathways and fast pyrolysis mechanisms have been reported for the production of valuable chemicals.^19^, ^20^

Despite significant progress over the last 30 years, a fundamental understanding of fast pyrolysis chemistry, that is, the key mechanistic details that lead to the formation of fast pyrolysis bio‐oil, is still lacking for a number of reasons: (i) the identification of chemical reactions based on the conversion of model compounds often leads to oversimplified degradation schemes; (ii) the inability of analytical techniques to describe bio‐oil immediately, fully and unequivocally; and (iii) the control of the pyrolysis regime is often impractical. In the study of degradation patterns, we cannot avoid isotopic spectroscopic techniques. Indeed, the use of non‐radioactive isotopes as tracers has been instrumental to provide further details on fragmentation mechanisms by allowing the distinction between intra‐ and inter‐molecular reactions and the quantitative assessment of the conversion of specific individual carbon atoms (i.e., ^13^C) in a molecule into other products, to mention a few examples.

To further current knowledge of the primary mechanisms of biomass “fast” pyrolysis, we propose an analytical procedure to assess and quantify the levels of “primary” products under controlled “fast” conditions. ^13^C‐enriched materials in conjunction with spectroscopic techniques are used to provide a more representative and quantitative description of bio‐oils. This study confirms and clarifies the general degradation scheme for biomass fast pyrolysis by providing a quantitative insight.

## Results and Discussion

### Characterization of raw materials

**Scheme 1:**
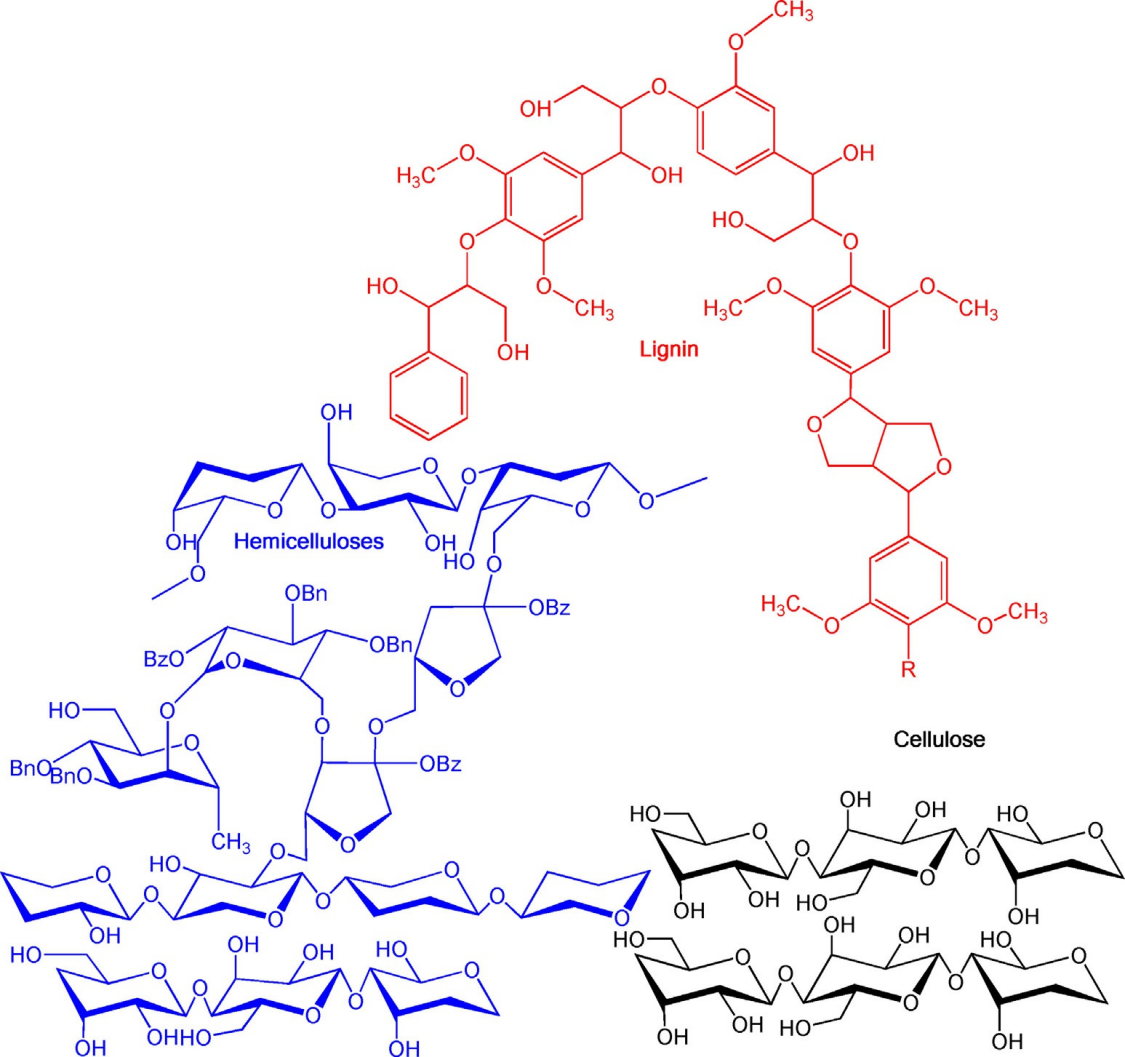
Illustration of potential lignocellulosic biomass fractions extracted from *Zea Mays*.

Unlabeled and ^13^C‐labeled leaves from natural *Zea Mays* grown under controlled conditions are composed of cellulose, hemicelluloses and lignin distributed evenly with 30–38 wt % of glucan, 23–25 wt % of xylan and 20–26 wt % of klason lignin.^21^ The main blocks were extracted according to classical methods. Cellulosic and hemicellulosic fractions were obtained using the classical method of a two‐step sulfur‐free soda pulping with sodium boron hydride in the first step to protect soluble hemicelluloses^22^ followed by further purification through selective bleaching and extraction steps to separate cellulose from hemicelluloses. Lignin was isolated from the black liquor obtained by two‐step sulfur‐free soda pulping adopted in a slightly modified form from Nadji et al.^23^ From the sugar composition (see the Supporting Information, Table S1) and details of extraction techniques (more details provided in Figure S1), a representation of the lignocellulosic composition of *Zea Mays* is proposed (Scheme 1). Notably, the broad chemical composition of the hemicelluloses that contain 55–62 % of xylose, 22–25 % arabinose, and 8–9 % of galactose agrees with previous results.^21^, ^24^

The extracted biopolymers displayed a low degree of purity of 58–59 % for cellulose, 48–50 % for hemicelluloses and 40–47 % for lignins, and all fractions contain different levels of impurities. For example, in the hemicellulosic fraction, 2.1–7.8 wt % of glucose remains with some lignin fragments and inorganics. The cellulosic fraction contains a substantial amount of xylan at 22.9 wt %. For the technical lignin, it is established that a significant fraction of sugars remain within the material as not all linkages of the lignin–carbohydrate complex are broken,^25^ and sugar levels reach 3.2 wt %.^26^

With respect to the presence of inorganics, semi‐quantification of the major elements by using SEM with energy‐dispersive X‐ray spectroscopy (EDS) revealed the ash composition within raw and technical materials (Table S2). The inorganic fraction of cellulose is composed mainly of Si, whereas Na and Ca make up that of hemicelluloses and Si and Na that of lignin. If the presence of some of these inorganic elements can be explained by the natural composition of the original plant, for example, *Zea Mays*, for which the inorganic matter is mostly composed of K and Ca, high levels of Na in both hemicelluloses and lignin could originate from salts contained in solutions or solvents used to extract or precipitate technical materials.^27^

The ultimate analysis of individual materials (Table 1) has allowed the deduction of general elemental formulae (Table 2). Their comparison with previous results indicates that the chemistry of the technical biopolymers differs from that of native constituents because of the presence of residual components. The chemical extraction had a substantial impact on the chemical composition of the lignin that sees its chemical structure altered significantly. Indeed, during the acid‐precipitation process for soda lignins, phenolic hydroxy groups are lost and condensation reactions are favored with the formation of carboxyl groups.^26^

**Table 1:** Ultimate analysis of unlabeled and extracted materials [wt %].

Feedstock	C	H	N	S	O+ash^[a]^	H/C
maize	41.6	5.65	1.31	0.06	51.4	0.14
cellulose	42.8	6.38	0	0	50.9	0.15
hemicelluloses	34.6	5.35	0.75	0	59.3	0.16
lignin	49.9	6.08	1.43	0	42.6	0.12

[a] Obtained by difference.

**Table 2:** General chemical formulae.

	Cellulose	Hemicelluloses	Lignin
this study	C_5.6_H_10_O_5_	C_5_H_9_O_6.5_	C_8_H_11_O_5_
previous reports		C_5.2_H_9.7_O_5_ 28	C_10.2_H_12.2_O_3.8_N_0.2_ 7
general	(C_6_H_10_O_5_)_*n*_ 29	(C_5_H_8_O_4_)_*n*_ and (C_6_H_10_O_5_)_*n*_ 30	

### Identification of pyrolysis products by pyrolysis GC‐MS

**Figure 1:**
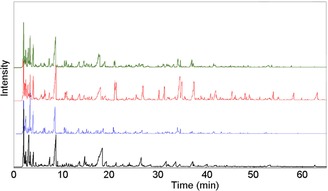
Chromatogram (GC–FID) of the products of the pyrolysis of extracted biopolymers from *Zea Mays*: — mixture; — lignin; — hemicelluloses; — cellulose.

The detection (Figure 1) and identification of organics by using pyrolysis (Py) GC‐MS (Figure S2) was useful to reveal some clear thermal and structural differences between technical biopolymers.

The detection of some typical components confirmed the botanical origin of the material, *Zea Mays*, as a grass. For example, the detection of ribofuranoside, pyranose and furanose compounds (Table S3) confirms the highly heterogeneous nature of hemicelluloses in grasses, which have an arabinoxylan structure.^31^ For lignin, the detection of aromatic components: phenols (e.g., 2‐methylphenol, 4‐dimethylphenol, 4‐ethylphenol), guaiacols (e.g., 4‐methylguaiacol (creosol), 4‐ethylguaiacol, *p*‐vinylguaiacol) and syringols (e.g., 2,6‐dimethoxy‐4,2‐propenylphenol; Table S3) confirms the presence of *p*‐hydroxyphenyl (H), guaiacyl (G), and syringyl (S) phenylpropanoid units within the original material. As expected, a number of sugars (e.g., 1,4:3,6‐dianhydroglucopyranose) were detected in the technical lignin, which confirms the intimate bonding between carbohydrates and lignin and the difficulty to separate them chemically.

Within a molecular range of 25–300 Da, the number of chemicals detected and identified is low. Indeed, the detection capability of GC techniques is limited by the volatility of the products and the heaviest compounds, such as oligomers, are not analyzed. To solve this technical issue, alternative chromatographic conditions (e.g., different columns) or techniques (e.g., gel‐permeation chromatography; GPC) must be used.^7^ In this study, we exploit the performance of liquid‐state NMR spectroscopy to assess the chemical composition of the whole bio‐oil, which is certainly less specific in terms of organics identification but more representative of its chemical composition.

### Nature of primary reactions

There is no unanimous consensus in the naming and listing of the number of degradation stages, which is a direct consequence of the complex character of fast pyrolysis; but researchers tend to agree that the thermal degradation of lignocellulose occurs through a series of primary, secondary and tertiary multi‐phase chemical reactions and are transformed into stable organic vapors and aerosols, carbonaceous residue and permanent gases.^32^

**Figure 2:**
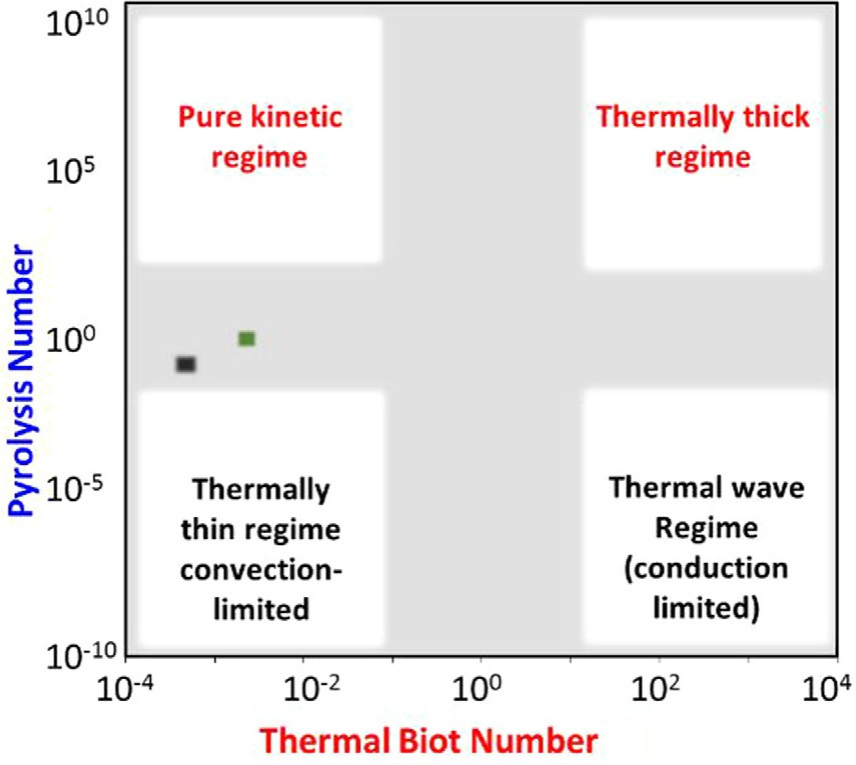
Mapping of pyrolysis regimes according to heat transport. Adapted from Ref. 33. Heat transport map for the pyroprobe (▪) and the microreactor (▪) at approximately 550 °C.

The overall degradation scheme of biomass “fast” pyrolysis can be seen as a combination of parallel and competing reactions, the occurrence and dominance of which is feedstock‐ and process‐related. The pyrolysis regime obeys the thermodynamic laws of transport (mode) and transfer (limitations), which are controlled mainly by the design of the reactor and the feedstock preparation. Researchers have mapped the different pyrolysis regimes according to characteristic times (Figure 2). The use of these characteristic times that illustrate the predominance of times between internal conduction to the external convection (thermal Biot number; Biot nb), between chemical reaction and internal conduction (internal pyrolysis number; Py′) or external convection (Darcy number; Da, or external pyrolysis number; Py′′) have permitted the boundaries to be defined. As a result, the pyrolysis regime must be controlled to allow the deduction of biomass degradation patterns in real‐world reactors and under “fast” conditions.

**Scheme 2:**
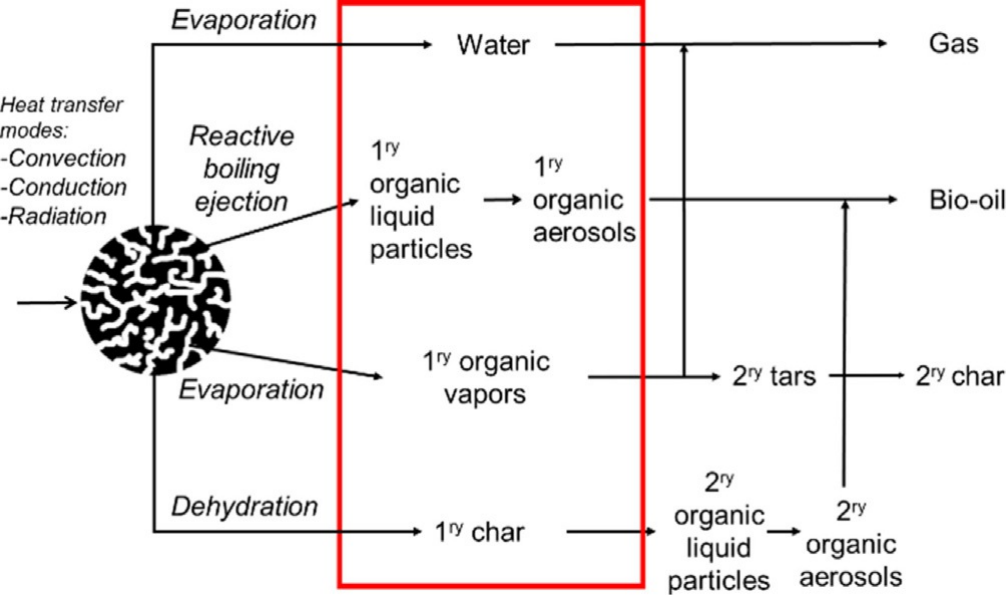
Intra‐ and extra‐particle mass and heat transport events.

In this study, two fixed‐bed reactors with distinct designs were used to transform technical biopolymers. The non‐dimensional numbers, Biot and pyrolysis numbers, were estimated, which permitted actual pyrolysis modes to be specified within experiments for the same characteristic length of the biomass particles. The values that correspond to the internal heat transport number, Py and thermal Biot number results were placed in the heat transport map (Figure 2). Pyrolysis is not isothermal in both the Pyroprobe and microreactor, and heat transport differs by one order of magnitude with the clear occurrence of heat and mass transfer limitations. This result is not surprising if we look at the major advancements that have been made recently in the development of experimental microreactors to address temperature gradient^33^ and temporal mismatch,^34^ which interfere with reaction kinetics. In the study of the primary reactions, it is important to prevent or limit the occurrence of any secondary reactions. In general, solid particles and volatiles that spend a short residence time (<1 s) in the hot zone are classified as “primary products” and result mainly from the fragmentation and shrinkage of particles. These primary products have different physical states: aerosols, vapors and/or gas for volatiles and solids. If they are retained in the hot zone, volatiles and residual solids undergo secondary reactions to result in the formation of secondary products (Scheme 2). These secondary reactions are typically categorized as heterogeneous gas–solid reactions and homogeneous gas‐phase reactions. The heterogeneous reactions include intra‐ and inter‐particle reactions between solid (unconverted biomass and/or char) and gas or liquid and gas, which result in secondary char and low‐molecular‐weight volatiles.^35^

**Figure 3:**
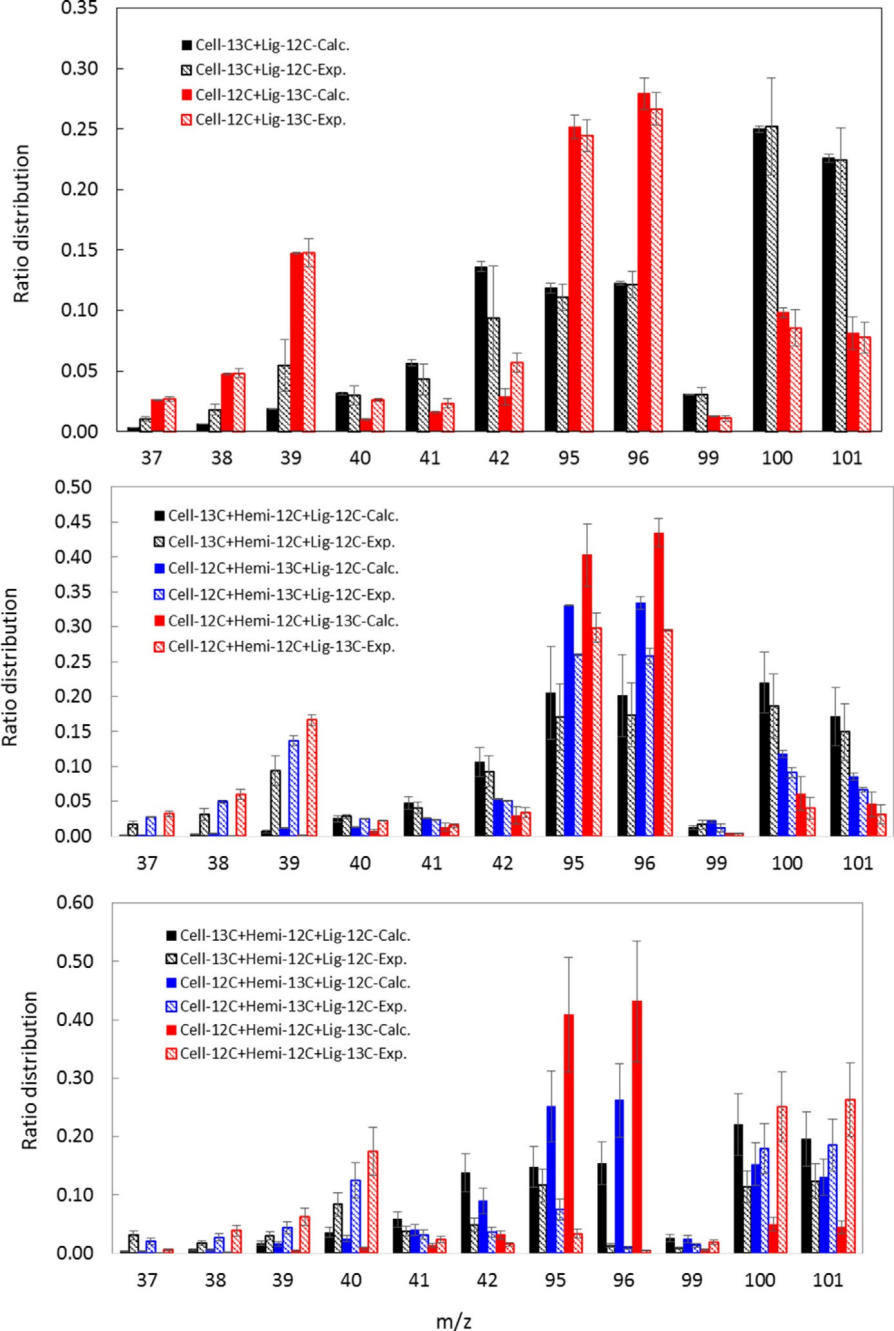
Confirmation of the product identity (furfural) and lack of scrambling by comparing experimental and predicted MS fragmentation patterns (ratio distribution vs. *m*/*z*) of FP products from mixtures of unlabeled cellulose (Cell‐^12^C), hemicelluloses (Hemi‐^12^C) and lignin (Lig‐^12^C) and ^13^C‐enriched cellulose (Cell‐^13^C), hemicelluloses (Hemi‐^13^C) and lignin (Lig‐^13^C). a) Mixture of cellulose and lignin processed by using Py‐GC‐MS; b) and c) Mixtures of cellulose, hemicelluloses and lignin processed, respectively, by using Py‐GC‐MS and the microreactor. Calc.=calculated; Exp.=experimental.

Although it has been demonstrated that it would be impossible to prevent any intra‐particle secondary reactions, it is, however, technically possible to control the number of extra‐particle secondary reactions by limiting the volatile residence time within the hot zone. To assess the impact of extra‐particle residence time of volatiles on chemical reactions, an isotope‐labeling approach with non‐radioactive materials and MS was used. Fragment re‐combinations between primary volatiles species and, more specifically, the modes of initial C−C bond breakage within the biopolymers could thus be studied.^36^ Detailed analyses of MS fragmentation patterns for furfural (Figures S2 and S3, Tables S4 and S5) produced through primary reactions^37^ from the pyrolysis of mixtures between unlabeled and ^13^C‐enriched materials using Py‐GC‐MS and the microreactor are shown in Figure 3 a–c. In the case of the mixed cellulose and lignin preparation analyzed by using Py‐GC‐MS, the good match between the experimental fragmentation and predicted patterns confirms the absence of carbon scrambling during the primary fast pyrolysis stage, which indicates the dominance and uni‐molecular character of intramolecular rearrangements. In the processing of the three‐biopolymer mixture, the difference between the calculated and experimental values became noticeable (Figure 3 b). This could result in a less predictable conversion because of the increasing complexity of reactions if hemicelluloses are added.

Discrepancies between experimental and calculated values became more prominent if the three‐biopolymer mixture was processed by using the microreactor (Figure 3 c). The use of the tubular microreactor with a long volatile residence of 1.8 s (vs. an order of milliseconds for the pyroprobe) had an adverse impact on the presence of secondary reactions. This is best represented by comparing the distribution ratios for *m*/*z* 95 and 96 (Figures 3 a–c), in which furfural production is decreased significantly if volatiles are exposed to longer residence times in the hot zone. Overall, the results suggest the occurrence of chemical interactions if hemicelluloses are added and the increase of the volatiles residence time affect the chemistry of primary reactions.

### Micropyrolysis of extracted biopolymers and their mixtures

**Figure 4:**
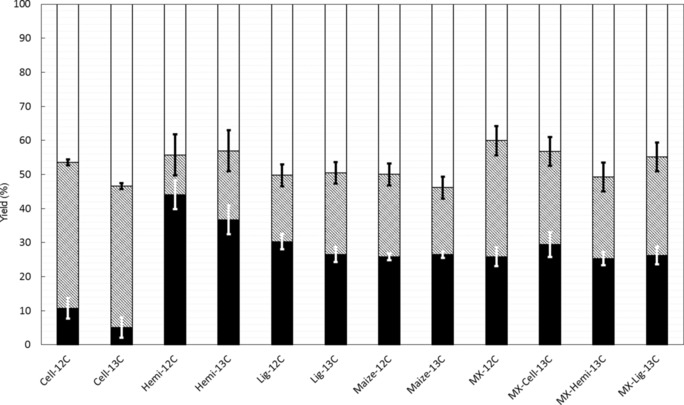
Yield of fast pyrolysis products for 550 °C: [▪] char yield; [▪+□] volatiles yield; [▪] total GC‐detectable product yield; [□] undetected product yield obtained by difference.

Each extracted biopolymer and their mixtures were fast pyrolyzed at 550 °C by using a conventional tubular reactor (Figure S4). As expected, the extracted cellulose displayed the lowest char yield (Figure 4). The solid residue is often attributed to the formation of “primary char” that results from the dehydration and charring processes of solid polymers and to the formation of “secondary char”, which results from polymerization reactions between volatile compounds.^35^ These polymerization reactions between volatile compounds are maximized if the volatiles are exposed to extensive heterogeneous residence times (i.e., solid–volatiles residence time).^38^ During the pyrolysis process, the volatiles were removed continuously and immediately from the hot zone at 550 °C and only had a homogeneous residence time (i.e., volatiles residence time) of less than 1.8 s. The fast pyrolysis of lignin led to approximately 28.4 wt % char yield in the range of those obtained from the conversion of raw biomass and mixtures (26.1 and 27.0 wt %, respectively). Although the presence of lignin has been reported to be the origin of char,^39^ its transformation led to lower char yields than those that result from the conversion of hemicelluloses (Figure 4). This can be explained by the presence of sugar impurities that may have inhibited the formation of char and facilitated the devolatilization of lignin and also by the aromatic character and heterogeneous nature of the hemicelluloses used in this study.

### Product distribution obtained by using GC‐MS

A portion of the fast pyrolysis bio‐oil could be analyzed by using off‐line GC‐MS. In total, 142 pyrolysis products were identified and 55 quantified (Table S6). These compounds have been lumped into chemical families according different classifications. A preliminary classification, in which these organics are grouped according their non‐aromatic, heterocyclic and aromatic character (Figure S5), indicates that most of the products detected by using GC‐MS (non‐aromatic and carbohydrates) have a non‐aromatic character.

**Figure 5:**
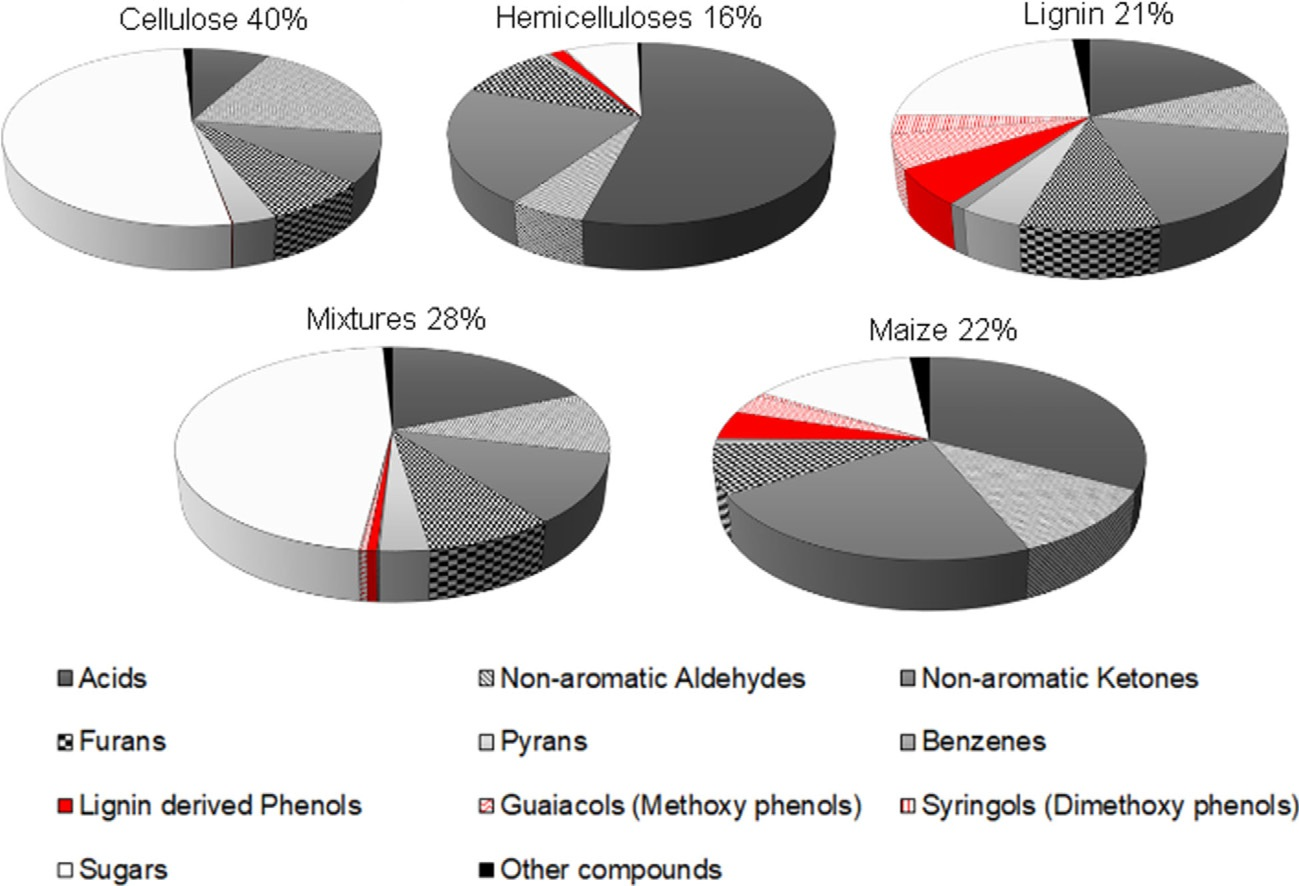
Relative proportions [%] of the important fractions in bio‐oil.

Refined degradation patterns can be obtained by adopting a detailed classification of organic compounds (Figure 5). The concentration of detected products differs according the nature of the biopolymer processed. The highest levels, up to 40 wt %, were detected by using GC for cellulose, which decreased to 16 and 21 wt % for hemicelluloses and lignin, respectively. The carbohydrate character of cellulose‐derived bio‐oil was confirmed with the detection of 20.75 wt % sugars. The highest level of acids (8.56 wt %) was found for the hemicelluloses‐derived bio‐oils, which indicates that the hemicellulosic fraction of biomass should be at the origin of the acidic character of bio‐oils because of uronic acid groups present in hemicelluloses.^30^ Equivalent amounts of acids and sugars, 3.87 and 4.91 wt %, within lignin‐derived liquids confirm a less acidic character than that of hemicellulosic liquids. In particular, the high concentrations of short‐chain acids (e.g., acetic and propionic acids levels of 4.8 and 3.8 wt % detected for hemicelluloses‐derived liquids vs. 1.8 and 2.1 wt % for the lignin‐derived liquids) have a catalytic effect on the oligomerization of phenolic compounds. This has been demonstrated experimentally in the presence of acetic acid,^7^ whereas the role of propionic acid is still unknown. Notably, only the lignin‐derived liquids contained a monomeric phenol fraction derived from the hydroxy‐ and methoxy‐substituted phenylpropane units.^40^

### Cellulose degradation patterns

Despite the vast number of cellulose degradation schemes available, a common pattern has been identified and can be summarized as follows: (i) depolymerization of cellulose into glucose through transglycosylation/retro‐aldol condensation (intramolecular rearrangement of the monomeric units); (ii) β‐elimination with the production of levoglucosan, which is further degraded into hydroxyacetaldehyde by (iii) ring fragmentation.^41^

The detection of high concentrations of levoglucosan (LG; 20.85 wt %) and hydroxyacetaldehyde (HA; 5.32 wt %) confirms the preponderance of the β‐elimination mechanism in cellulose de‐construction under fast pyrolysis. The level of LG remains much lower than that recorded if the reactor design allows the preservation of the molten phase for longer times,^15^ which confirms that the selectivity towards LG can be increased by minimizing homogeneous secondary reactions and increasing the heat flux density. This loss of LG selectivity was beneficial to the formation of other anhydrosugars (e.g., 1,5‐anhydro‐β‐d‐arabinofuranose, 1,5‐anhydro‐β‐d‐xylofuranose, 1,6‐anhydro‐α‐d‐galactofuranose, 1,4:3,6‐dianhydro‐α‐d‐glucopyranose) with an average of 20.8 wt %, which indicates that secondary pyrolysis is abundant in this conventional tubular microreactor.

However, high levels of smaller products, such as furans (e.g., 2.83 wt % 5*‐*hydroxymethylfurfural (5‐HMF) and 0.94 wt % furfural) and small oxygenates (e.g., 1.13 wt % acetic acid and 1.47 wt % propionic acid), indicate that the ring fragmentation of cellulose leads directly to a large portion of furanic products^37^ and that this degradation pathway (iii) is favored over elimination reactions that lead to pyrans (e.g., 1.23 wt % of 3‐hydroxy‐5,6‐dihydro‐(4*H*)‐pyran‐4‐one) and anhydrosugars.

### Hemicellulose degradation patterns

The main degradation events for the pyrolysis of hemicelluloses are, in general, the least investigated among major lignocellulosic fractions and can be summarized as follows: (i) depolymerization of the xylan fraction into xylose by the breakdown of the glycosidic bond, (ii) production of anhydrosugars and pyran compounds by rearrangement, (iii) competing reactions between the ring breakage of the anhydrosugars and pyrans into light oxygenates (e.g., carboxylic acids, aldehydes and furans that contain 1–5 C atoms).

If we consider the GC‐MS analyses, the selection of pure standards based on the major degradation of cellulose and lignin did not permit the main degradation trends to be described and represented only 16 wt % of organics within the condensates (Figure 5). The high levels of acetic and propionic acids and hydroxypropanone (4.8, 3.8 and 0.9–3.3 wt %) are still an indication that the hemicellulosic fraction contains a number of ring units that are broken easily into light oxygenates and that the acetyl groups that are usually sensitive to alkaline hot extraction^42^ are retained in the structure. In the list of identified compounds, a number of five‐carbon heterocyclics not detected in the case of cellulose and lignin support the idea that the thermal processing of hemicellulosic fraction may result in a new range of chemicals. The transformation of these technical hemicelluloses did not lead to LG, which indicates that the cellulosic fraction 2.1–7.8 wt % (Table S1) that is left after extraction could have little impact on the final product distribution.

If compared to previous work on the fast pyrolysis of extracted hemicelluloses,^6^ considerable deviations in the composition of condensates were observed. This is attributed mainly to the different botanical origin of the feedstock from which the hemicelluloses were extracted. Indeed, hemicelluloses derived from *Zea Mays* leaves contained less xylose, 31.1–31.6 wt % on average (Table S1), than that from switchgrass (66.2 wt %).^6^ We may also expect that the type of isolation and/or purification methods also influenced this product distribution. The clearest difference was the detection of propionic acid instead of formic acid from *Zea Mays*. The disparity between the yields and the nature of the carboxylic acids was attributed to the different heat transfer and reaction time scales used in these studies, a heating rate of 452 °C s^−1^ in our case versus the claimed rapid heating rate of >2000 °C s^−1^.^37^ Faster heating rates promote the rupture of the H_2_C−COOH linkage.

Among the levels detected, carboxylic acids are the most abundant, with an average amount of 8.6 wt %, followed by 3.4 wt % of non‐aromatic ketones, 1.4 wt % of furans, 1.2 wt % of sugars, and 0.87 wt % of non‐aromatic aldehydes. Substantial amounts of ketones and furans were also detected if extracted hemicelluloses were converted in a tubular fixed‐bed reactor,^43^, ^44^ and furfural was the most abundant furanic compound. The high concentrations of acetic acid detected confirmed that hemicelluloses are the biopolymers that produce the most of this acid.

### Lignin degradation patterns

On undergoing fast pyrolysis, lignin is converted into both unstable and stable products through two competitive reactions: (i) the thermal cleavage of inter‐unit or alkyl linkages and (ii) char formation. The primary condensates are composed mainly of monomeric phenolic compounds.^7^ This was confirmed by the detection of a range of phenols (e.g., 0.18 wt % of phenol, 0.60 wt % of 4‐vinylphenol), methoxyphenols (e.g., 0.17 wt % of guaiacol, 0.52 wt % of 4‐vinylguaiacol, 0.14 wt % of vanillin) and dimethoxyphenols (e.g., 0.17 wt % of syringol, 0.13 wt % of 4‐vinylsyringol, 0.14 wt % of acetosyringol). In this study, the relative ratio of phenol (P), guaiacol (G) and syringol (S) units was 1.1:1.0:0.6, which is comparable to the original ^13^C‐enriched *Zea Mays* ratio of 2.1:1.0:0.9. This was determined by approximating the ^13^C liquid‐state cross‐polarization magic‐angle spinning (CP‐MAS) NMR spectrum,^21^ which indicated that a large proportion of monolignol units was not fully released. This confirms the liberation of oligomers, most probably dimers^7^ that contain two phenols units connected by a 5,5‐biphenyl type linkage, which is the most recalcitrant towards thermal cleavage.^45^

In addition to phenylpropane units of various degrees of methoxylation, the lignin fraction of the native *Zea mays* possesses numerous side chains with different types of carbon atoms (Cα, Cβ, C_Y_) and oxygenated groups such as alcohol, carbonyl and carboxylic acid functions.^21^ If pyrolyzed, these side chains generate light‐oxygenate carboxylic acids (e.g., 1.8 wt % of acetic acid, 2.1 wt % of propionic acid).

### Degradation patterns of raw biomass and mixtures of three biopolymers

The distribution of the major pyrolysis products from *Zea mays* corresponds to that depicted for each biopolymer, that is, high levels of light oxygenates (7.0 wt % of carboxylic acids, 2.5 wt % of non‐aromatic aldehydes and 4.1 wt % of non‐aromatic ketones in the bio‐oil produced), a common trend for all lignocellulosic fractions. A similar product distribution in light oxygenates was obtained for the mixture (5.1 wt % of carboxylic acids, 2.8 wt % of non‐aromatic aldehydes and 3.0 wt % of non‐aromatic ketones in bio‐oil). Some furans and pyrans, 1.7 and 0.13 wt %, respectively, were also produced and are attributed to the degradation of cellulose. Only a few lignin‐derived compounds (e.g., 4‐vinylphenol and 4‐vinylguaiacol) were detected at a total concentration of 1.0 wt % and derived from technical hemicelluloses and lignin transformations. This low production of lignin‐derived products (0.2 wt %) was also measured if hemicelluloses, cellulose and lignin were mixed and converted. A striking difference in the product distribution is the amount of sugars produced: 3.2 wt % for the native biomass versus 13.5 wt % for mixtures.

Another distinctive feature between the conversion of biomass and mixtures of technical biopolymers is the level of hydroquinone (3.1 wt %), which was detected at a low level for the mixture (0.1 wt %), and yet it appears to be a common compound detected on many occasions in the study of the pyrolysis of biomass^9^, ^46^ and technical lignin.^47^ Furthermore, there was an unexpected low level of levoglucosan: 0.6 wt % for the biomass versus 10.4 wt % on average for mixtures. Finally, the thermal conversion of mixtures produced a low amount of lignin‐derived compounds: 0.2 versus 1.0 wt % for biomass.

The ratio of C, H, and L was based on compositional analyses of the same feedstock, *Zea Mays*, found in the literature that indicates that the hemicellulosic fraction makes up the largest part of the feedstock. Although we cannot, therefore, guarantee the perfect reproduction of the lignocellulosic composition of the native material, the comparison of product yields between the raw biomass and mixture confirms the key role of linkages and additives (i.e., extractives and inorganics) during pyrolysis. The association of those components prevents the efficient release of monolignols and affects the degradation patterns of cellulose greatly. For instance, the formation of hydroquinone combined with changes in the pyrolysis degradation patterns of all biopolymers is an indicator that the presence of a “radical scavenger”,^48^ such as hydroquinone, could interfere and reassign the dominance of fast pyrolysis degradation modes (i.e., ionic and nonionic modes).

If we consider the yields of individual key products for bio‐oils derived from mixtures, the production of cellulose‐derived products (e.g., glycoaldehyde, levoglucosan, 5‐HMF) was not enhanced and that of lignin‐derived products was substantially suppressed. These results indicate that the reported beneficial effect of the presence of lignin on cellulose degradation and vice versa during primary pyrolysis^35^ was inhibited by the presence of hemicelluloses. These results indicate that the mechanistic explanation suggested by Hosoya et al.^35^ that the “polymerization of anhydrosugars is inhibited by the lignin‐derived volatile products” to the benefit of oxygenated five‐carbon heterocycles production is unlikely to happen under these fast pyrolysis conditions. However, these results confirmed the competition between the cleavage of glycosidic and C−C bonds, which has now been reported many times.^35^

### Quantitative liquid‐state ^13^C NMR spectroscopy

Very recently, liquid NMR spectroscopy techniques have become used widely for biomass pyrolysis product analysis, as these methods provide an accurate view of the chemical composition of condensates by mapping the overall distribution of the functional groups. This reveals important changes in the chemistry of pyrolysis according to pyrolysis regimes in terms of non‐dimensional characteristic numbers.^18^ However, if we consider both the small amount of condensates that can be collected under controlled “fast” pyrolysis and the low natural abundance of the ^13^C isotope, the use of ^13^C‐enriched substrates has permitted their analysis by increasing the magnitude of the ^13^C NMR resonance signals (Figure S6).^21^ As the extracted biopolymers used in this study have a low purity, we noticed further resonance lines derived from the impurities (e.g., the presence of xylans in the cellulose‐derived condensates, glucan in hemicellulosic‐based liquids and sugars in the liquid produced from impure technical lignin) in the NMR spectra.^49^ This was taken into consideration when we attributed the relevant chemical shift regions to the corresponding chemical functions (Figure S6) according to chemical shift ranges proposed in previous studies (Table 3).

**Table 3:** Ranges of chemical shifts *δ* in the ^13^C NMR spectra.

*δ* [ppm]	Groups	Ref.
215–180	ketones, aldehydes	55
180–163	esters, carboxylic acids	55
163–110:	aromatic (general)	
•125–112	aromatic compounds (guaiacyl compounds)	
•112–110	aromatic compounds (syringyl compounds)	
110–84	carbohydrate‐type carbon atoms	56, 57
84–54	methoxy, hydroxy bond compounds (R−CH_2_−O−R, R−O−CH_3_)	
54–1:	primary, secondary, tertiary and quaternary alkyl carbon atoms	
•34–24	most of secondary and tertiary alkyl carbon atoms	
•24–6	most of primary and some secondary alkyl carbon atoms	

**Figure 6:**
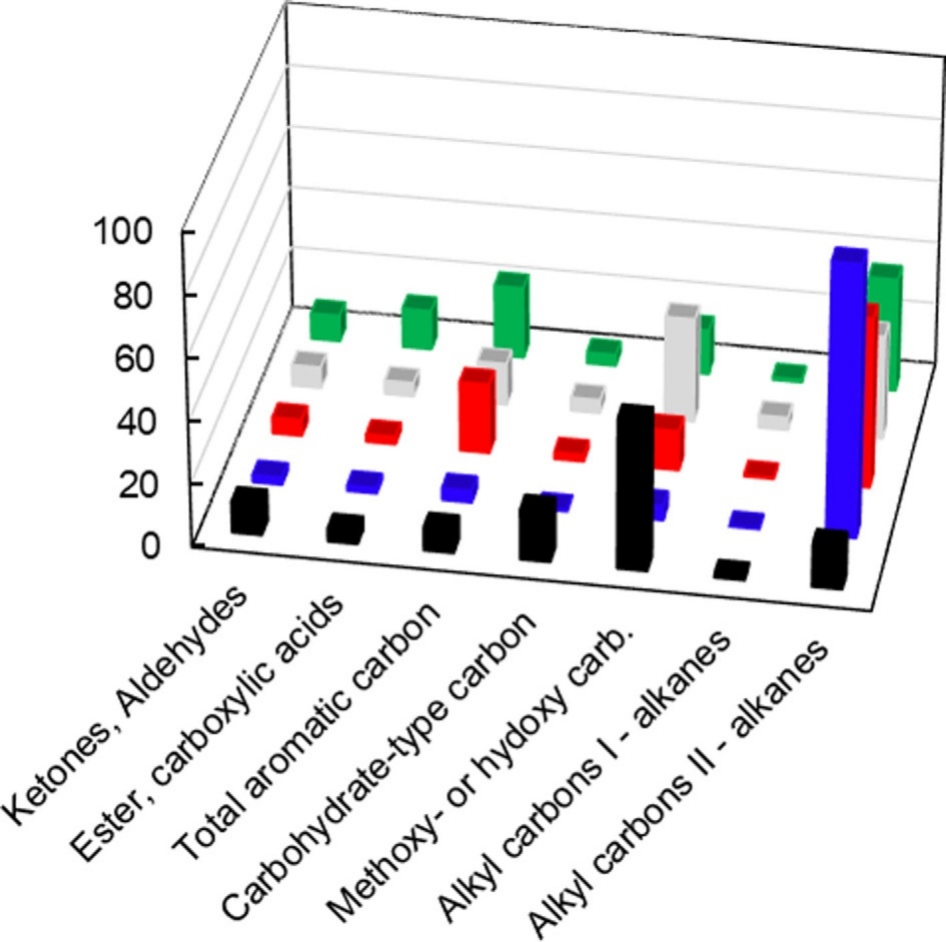
Relative portions [%] of chemical groups within enriched bio‐oils obtained by using liquid‐state ^13^C NMR spectroscopy: [▪] Cell‐^13^C; [▪] Hemi‐^13^C; [▪] Lig‐^13^C; [▪] MX^13^C (MX=mixture); [▪] Maize‐^13^C. Alkyl carbons I and II refer to primary and secondary alkyl carbon atoms, respectively.

The relative distribution of functional groups within bio‐oil determined by using quantitative liquid ^13^C NMR spectroscopy (Figure 6 and Table S7) indicates that methoxy or hydroxy carbon atoms prevail in the cellulose‐ and mixture‐derived ^13^C‐enriched liquids, which confirms that a large proportion of the aliphatic C−O functions within condensates is related to the carbohydrate fraction in biomass. Secondary alkyl carbon atoms represent the largest proportion in lignin‐derived condensates because of the highly branched character of the polyphenolic structure with side chains that contain secondary carbon atoms.^27^ The large amount of aliphatic C−C bonds combined with the lack of carbon scrambling confirms that this type of linkage was not cleaved during the primary fast pyrolysis reactions and refutes any mechanistic suggestions of the participation of these groups in the formation of aromatics. A significant amount of these secondary carbon atoms was also found in hemicellulose‐derived liquids; a tempting interpretation would be to suggest hemicelluloses as a potential source of long carbon chains. The overall chemical composition of bio‐oils produced from the fast pyrolysis of raw *Zea Mays* was found to best match that of lignin‐derived liquids (Table S8), which suggests that the chemical composition of the volatiles generated during the fast pyrolysis of lignocellulose is affected significantly by the presence of the polyphenolic biopolymers.

### Carbon source issue from fast pyrolysis reactions

To confirm the origin of functional groups according to the lignocellulosic composition, the co‐pyrolysis of a ^13^C‐enriched polymer mixed with two unlabeled polymers was performed. We used the quantitative results obtained from liquid‐state ^13^C NMR spectroscopy to determine the origins of the carbon by distinguishing carefully between contributions from the ^13^C NMR signals from the ^13^C‐enriched and unlabeled materials. More details can be found in the Supporting Information.

**Figure 7:**
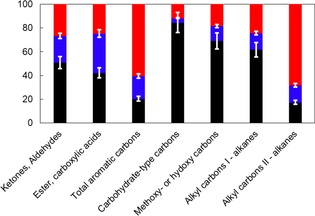
Carbon source of chemical families according to the extracted biopolymers: [▪] cellulose; [▪] hemicelluloses; [▪] lignin.

Most of the carbohydrate products are depicted in Figure 7, which shows that functional groups such as methoxy/hydroxy carbon atoms and alkyl primary carbon atoms are derived mostly from cellulose.

**Figure 8:**
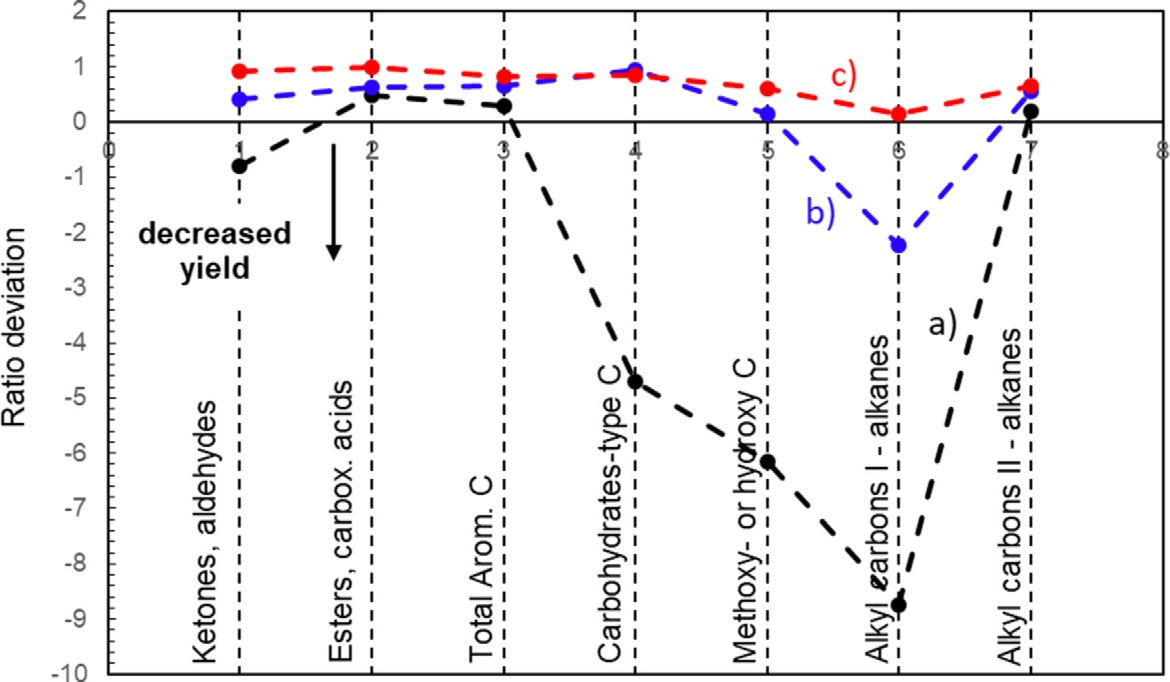
Ratio deviation between experimental and theoretical yields of organic groups for a) cellulose, b) hemicelluloses and c) lignin.

Ratio deviations (Figure 8) were calculated from experimental and estimated values of ^13^C moles per gram of condensates. The values were obtained from the experimental results for each technical component pyrolysis weighted by their mass percentage proportion within mixtures by assuming that there is no interaction between these biopolymers. The difference between the experimental and estimated yields (i.e., the deviation from the *x* axis in Figure 8) indicates the extent of these interactions. The most significant deviations were obtained for cellulose‐derived products with substantially lower intensity for carbohydrate‐type carbon atoms and methoxy or hydroxy carbon atoms, which implies that the degradation pattern of cellulose is affected greatly by the presence of hemicelluloses and lignin. This result confirms the important role of cellulose‐hemicelluloses and cellulose‐lignin bonding ascribed to hydrogen^50^ and ether^51^ bonding, respectively. Conversely, the degradation patterns of hemicelluloses and lignin remained consistent, and the ratio deviation varied in a small range. In some cases, the detection of some chemical families (e.g., alkanes that contain primary alkyl carbon atoms) tends to increase slightly in the presence of cellulose.

## Conclusions

We compared the fast pyrolysis of technical cellulose, hemicelluloses, lignin and their mixtures in an attempt to provide a more explicit thermal degradation scheme for lignocellulosic material. The conversion of these biopolymers was achieved by using two different fast pyrolysis microreactors (i.e., pyroprobe and tubular microreactor) at 550 °C. The use of isotopic spectroscopy techniques was also explored to provide a quantitative view of the overall product distribution under fast pyrolysis conditions.

We combined the fast pyrolysis product distributions obtained from extracted unlabeled and ^13^C‐enriched cellulose, hemicelluloses and lignin to propose a degradation model to be developed for biomass fast pyrolysis in which the products were grouped according the original lignocellulosic distribution of the biomass. This new NMR spectroscopy approach that combines the overall analysis of bio‐oil by ^13^C NMR and isotope‐labeled starting materials will be useful to express global kinetic expressions. The comparison of the degradation patterns between native biomass and mixtures of extracted lignocellulosic biopolymers indicates a potential “inhibitor” role of catechols to drive the degradation modes.

Liquid ^13^C NMR spectroscopy, in association with the use of ^13^C‐enriched isotopic material, permitted the technical limitations related to the low natural abundance of ^13^C (1.1 %) to be overcome, which made the interpretation of the ^13^C NMR spectra much easier and more reliable. The applicability of our approach to widespread pyrolysis conditions needs to be tested. If successful, the use of labeled compounds combined with ^13^C NMR spectroscopy could become a useful tool to assess the stoichiometry of balanced chemical equations.

## Experimental Section

### Cellulose and hemicelluloses extraction

*Zea Mays* was subjected to a series of extractions (i.e., a cold ethanol extraction (0–4 °C), a Soxhlet extraction with ethanol/toluene (2:1, v/v) and a hot‐water extraction at 100 °C) before further extractions.

Cellulose and hemicelluloses were extracted by following the classical two‐step sulfur‐free soda pulping method proposed by Huisman et al.^22^ The first step consisted of the pretreatment of the washed biomass using a 10 % NaOH solution (10 % on dry matter basis at a solid/liquid ratio of 1:10 at 60 °C for 1 h) in the presence of a sodium boron hydride solution. The cellulosic solid was bleached by H_2_O_2_ (5 %), extracted with diluted (2 %) alkali, again bleached by H_2_O_2_ (5 %), washed and vacuum‐dried (at 40 °C). Its purity (after hydrolysis) was analyzed by using HPLC (Dionex HPAEC‐PAD).

The hemicellulosic fraction was obtained by precipitation of the supernatants from the first pre‐hydrolysis step using ethanol, followed by an acid hydrolysis and a final selective precipitation of the glucoronoarabinoxylans with water.

### Lignin extraction

The isolation of alkali‐solv lignin, more commonly Soda lignin, was done according to a two‐step sulfur‐free soda pulping method established by Nadji et al.^23^ with some modifications. The pulping stage consisted of heating a mixture of *Zea Mays*/liquor (Na_2_O) 4:1 (w/w) at approximately 120 °C for 1.5 h. The lignin was precipitated from the black liquor with concentrated formic acid to pH 3 and cooled overnight to 4 °C. The resulting precipitate (lignin) was isolated by centrifugation, washed twice with 10 % formic acid and three times with demineralized water to pH 5–6 and freeze‐dried.

### Feedstock characterization and preparation

Raw unlabeled and ^13^C‐enriched *Zea Mays* leaves (Maize‐^12^C and Maize‐^13^C) and unlabeled and ^13^C‐enriched cellulose (Cell‐^12^C and Cell‐^13^C), hemicelluloses (Hemi‐^12^C and Hemi‐^13^C) and lignin (Lig‐^12^C and Lig‐^13^C) extracted from *Zea mays* leaves were purchased from IsoLife (Wageningen, The Netherlands). The unlabeled feedstocks all displayed a natural abundance of ^13^C of less than 1.3 at %, whereas the labeled materials were enriched uniformly with a ^13^C content above 97 at %. Elemental analysis of individual feedstocks was performed by using a Flash 2000 Organic Elemental Analyzer (Thermo Fisher Scientific) with sulfanilamide as the standard (CE Instrument).

The particle size distribution was determined from SEM images to give a particle size of 50–150 μm.

Analysis of inorganic elements: Samples (i.e., raw materials and chars) were analyzed by using a Philips XL30 FEG ESEM scanning microscope combined with an Oxford Instruments INCAx EDS. A standard analysis protocol was applied. Samples were deposited onto double‐sided adhesive carbon mounting tabs and carbon coated by using an Emscope SC500 sputter coater. These analyses were utilized to provide particle size distribution, topographical and morphological images of particles and to semi‐quantify the composition of the major inorganic elements.

Preparation of feedstocks for Py‐GC‐MS: Mixtures of Cell‐^12^C+Lig‐^12^C, Cell‐^13^C+Lig‐^12^C and Cell‐^12^C+Lig‐^13^C were prepared according to a mass ratio of 7:3 by using a Sartorius microbalance (Model ME36S). This ratio was selected according a previous study that applied Technical Association of the Pulp and Paper Industry (TAPPI) standard methods (T264 om‐88, T211 om‐85) to determine the compositional analysis of corn stover with proportions (i.e., holocellulose, cellulose and lignin) determined gravimetrically. The lignocellulosic composition given was approximately 42.3 wt % of hemicelluloses (determined by difference), 37.0 wt % of cellulose and 13.0 wt % of lignin.^52^ The mixtures prepared from extracted materials were mixed for 4 h by using a roller mixer (Model SRT6D) at 60 rpm.

Preparation of feedstocks for the micro‐fast pyrolyzer: Raw unlabeled and enriched *Zea Mays* leaves were cryo‐milled in a cryogenic freezer/mill (Spec SamplePrep Model 6750) for 2 min per cycle. A cooling time of 15 min was set between the two milling cycles to avoid any overheating of the biomass.

Mixtures of Cell‐^12^C+Hemi‐^12^C+Lig‐^13^C, Cell‐^13^C+Hemi‐^12^C+Lig‐^12^C, Cell‐^12^C+Hemi‐^13^C+Lig‐^12^C, and Cell‐^12^C+Hemi‐^12^C+Lig‐^13^C were prepared with a mass ratio of 4:4.5:1.5 by using a Sartorius microbalance (Model ME36S) and roller mixed for 24 h before use.

### Py‐GC‐MS with flame ionization detection

The fast pyrolysis of individual components and mixtures was performed by using a CDS 5200 pyroprobe (CDS Analytical). The reactor consisted of an open quartz tube (25 mm length, 1.9 mm ID) that was inserted inside a heated probe. The quartz tube was heated electrically by using a Pt coil, which was calibrated according to the supplier specifications. The feedstock was placed into the open quartz tube (CDS Analytical) on the top of a quartz wool bed (CDS Analytical). A gas flow of He (pure He, 99.996 %, BOC) of 18 mL min^−1^ was maintained to remove the volatiles from the hot zone continuously. Before analysis, the volatiles passed through different isothermal zones: (i) the on‐line/off‐line valve oven to permit the heating and cooling of the interface zone, (ii) a trapping zone (Tenax‐TA pre‐column (PerkinElmer) set at 310 °C) that prevents any secondary reactions between volatiles and (iii) a heated transfer line (CDS Analytical) set at 310 °C connected to the chromatographic system.

For each experiment, a small amount of material (0.6–2 mg; Sartorius microbalance ME36S model) was introduced into the quartz tube and subjected to a heating rate of 452 °C s^−1^ to reach the desired pyrolysis temperature of 550 °C for 1.5 s. These conditions were selected to mimic the time–temperature history of a particle within a bubbling fluidized‐bed reactor.^53^ On‐line analysis of volatiles was done by using a PerkinElmer GC‐MS/FID (FID=flame ionization detection) system (Clarus 680‐Clarus 600S). Their separation was done by using an Elite 1701 column (30 m×0.25 mm×0.25 μm film thickness). He was used as the carrier gas at 15 mL min^−1^. The sample was injected at 275 °C using a split ratio of 1:50. The heating program of the oven was programmed as follows: heating from 45 to 280 °C at a rate of 2.5 °C min^−1^. Once separated, the organics were identified at *m*/*z* 30–300 Da. The MS spectra obtained were compared to the standard spectra of compounds found in the NIST library (2011). The identity of each compound was confirmed only if the fragmentation pattern with the detection of major *m*/*z* signals and intensity distribution matched the NIST spectrum. The order of separation was checked to according the retention time of pure standards found in a previous study using the same system.^54^

### Micro‐fast pyrolysis and volatiles condensation

The horizontal system consists of a quartz tube (length: 50 cm and internal diameter: 1 cm) heated by two independent horizontal furnaces. Both heated zones were controlled by using two independent thermocouples placed between the outer surface of the quartz tube and the heating element. The quartz boat that holds the feedstock (ca. 20 mg) was placed in the first zone of the tube at RT before the two heated zones. Once the boat had been placed and the furnace preheated to the desired temperature, the tube was flushed with pure N_2_ (Linde 5.0) set at a flow rate of 50 mL min^−1^ (ADM2000 Agilent flowmeter) under atmospheric pressure. The boat was introduced into the first heated zone by using a magnetic manipulator. Fast pyrolysis took place at a fixed temperature of 550 °C, and the sample was maintained in the zone for 70 s. After this required time, the boat was returned rapidly to its initial position. Volatiles (i.e., vapors and aerosols) passed through the second heated element set at 350 °C to prevent them from condensing on the tube wall before their collection. Condensable volatiles were quenched immediately by using a vapor‐trapping system (i.e., a pear shaped flask made of borosilicate glass with a glass cold finger) placed in a beaker that contained dry ice and acetone at around −50 °C. The permanent gases were allowed to exit through a cotton wool filter. Once the condensates had been collected, the trapping system was disconnected and sealed before further analyses. The boat was removed and weighed by using a Mettler Toledo XSE205 microbalance. All different parts of the setup were cleaned thoroughly with water/acetone and dried at 105 °C. The char yield was calculated directly, and the volatiles yield was determined by difference. The reproducibility of yields was achieved by pyrolyzing *Pinus Radiata* wood three times for which a standard deviation of 2.3 wt % for char yield was obtained.

### Condensates characterization by GC‐MS/FID

Once the beaker reached RT, the film of condensates deposited on the walls was recovered using 500 μL of an acetone solution that contained 209.48 μL mL^−1^ of fluoranthene (Sigma–Aldrich, 99 %) as an internal standard and an additional 100 μL of acetone (Sigma–Aldrich, ≥99.9 %). The identification and quantification of condensates was performed by using a HP 6890 Agilent GC system coupled to a HP 5972 mass spectrometer. The GC was equipped with a Varian VF 1701‐MS column (14 % cyanopropylphenyl/methylpolysiloxane, 60 m×0.25 mm dimension; 0.25 μm film thickness). The carrier gas used was He (Linde) at an initial flow rate of 1.3 mL min^−1^ and 225.7 kPa constant pressure. The injection was operated at 250 °C in a split mode (50:1) with a split flow of 66.1 mL min^−1^ and a total flow of 70.2 mL min^−1^. A second wash was applied to ensure the complete recovery of condensates with 100 μL of acetone that contained 2.263 mg mL^−1^ of internal standard (IS) fluoranthene and 1000 μL of pure acetone. This last sample was injected in a splitless mode. The GC column was heated initially at 45 °C for 4 min and then from 45 to 280 °C at 3 °C min^−1^ and held for 20 min. The quantification of compounds was performed by using a FID operated at 280 °C using a H_2_ flow rate of 40.0 mL min^−1^ and an air flow rate of 450.0 mL min^−1^. The detection was done using MS operated in the scan acquisition mode (19–550 amu). The MS source and MS quadruple temperatures were 230 and 150 °C, respectively. The identification and quantification of compounds was performed with the software OpenChrome using a home‐made library elaborated at vTI‐Institute of Wood Technology. The quantification of 55 compounds was done based on calibration curves. The reproducibility of the GC‐detectable content measurements was assessed by injecting the collected volatiles three times to result in a standard deviation of 0.2 and 0.3 wt % for the first and second wash, respectively.

### Quantitative ^13^C NMR spectroscopy

Liquid‐state ^13^C NMR spectroscopy were performed by using a Bruker Avance 3400 MHz for ^1^H NMR spectroscopy system. [D_6_]DMSO (99.8 %, deuteron) was used as the solvent and hexamethyldisiloxane (>98 %, Aldrich) as an internal standard. The condensates (ca. 15–20 mg) were collected directly from the glassware by adding 600 μL of an internal standard solution, the composition of which depended on the type of feedstock processed. For unlabeled bio‐oil samples, a volume of 600 μL internal solution HMDSO/[D_6_]DMSO 1:250 (w/w) was used. For the enriched bio‐oil sample, a solution of HMDSO/[D_6_]DMSO 1:30 (w/w) was used. NMR spectra were acquired by using inverse‐gated decoupling pulse sequences, 90° pulse angle and with a relaxation delay of 5.5 s (5×*T*1=*D*1) between pulses. To maintain a reasonable analysis time, a relaxation agent, chromium acetylacetonate (2.1 mg; Sigma–Aldrich, 99.99 %) was added to every NMR sample. The resulting experiments, however, still required several hours to obtain a sufficient signal‐to‐noise ratio to allow for the integration of spectra. If we consider the operating conditions and the good repeatability of the GC‐MS analysis performed on fast pyrolysis condensates of controlled and ^13^C‐enriched materials, no duplication of ^13^C NMR spectroscopy was performed. However, the accuracy of the NMR spectroscopy was determined by integrating the signal on a spectral width of 1 ppm in the frequency domain in which no signal was detected for each spectrum.

## Conflict of interest

*The authors declare no conflict of interest*.

## Supporting information

As a service to our authors and readers, this journal provides supporting information supplied by the authors. Such materials are peer reviewed and may be re‐organized for online delivery, but are not copy‐edited or typeset. Technical support issues arising from supporting information (other than missing files) should be addressed to the authors.

Supplementary
