# Supplementary material for: Quantitative Insights into the Fast Pyrolysis of Extracted Cellulose, Hemicelluloses, and Lignin
Source: ChemSusChem. 2017 Jul 25;10(16):3212–24. doi: 10.1002/cssc.201700984 (PMC5582602; doi:10.1002/cssc.201700984)
Supplement: Supplementary file 1 — Supplementary [file CSSC-10-3212-s001.pdf]

CHEMISTRY & SUSTAINABILITY
CHEMSUSCHEM
ENERGY & MATERIALS

Supporting Information

Quantitative Insights into the Fast Pyrolysis of Extracted Cellulose, Hemicelluloses, and Lignin

Marion Carrier,*[a] Michael Windt,[b] Bernhard Ziegler,[b] Jörn Appelt,[b] Bodo Saake,[c] Dietrich Meier,[b] and Anthony Bridgwater[a]

cssc_201700984_sm_miscellaneous_information.pdf

Table S1. Sugar contents (Glu: glucose; Ara: arabinose; Gal: galactose; Man: mannose; Rha: rhamnose; Xyl: xylose) within the carbohydrates fractions.

Sample	Glu[a]	Ara[a]	Gal[a]	Man[a]	Rha[a]	Xyl[a]	Lignin
Cell-^{12}C	59	2.7	0.6	n.d.	n.d.	22.9	4.2
Cell-^{13}C	58	2.3	0.4	n.d.	n.d.	20.1	4.4
Hemi-^{12}C	2.1	12.4	4.5	n.d.	n.d.	31.1	n.q.
Hemi-^{13}C	7.8	12.7	4.5	0.3	0.8	31.6	n.q.

n.d.: non-determined; n.q.: non-quantified

Figure S1. Strategy of extraction adapted from Huisman *et al.* [1] and Nadji *et al.* [2] by IsoLife, the supplier of extracted materials.

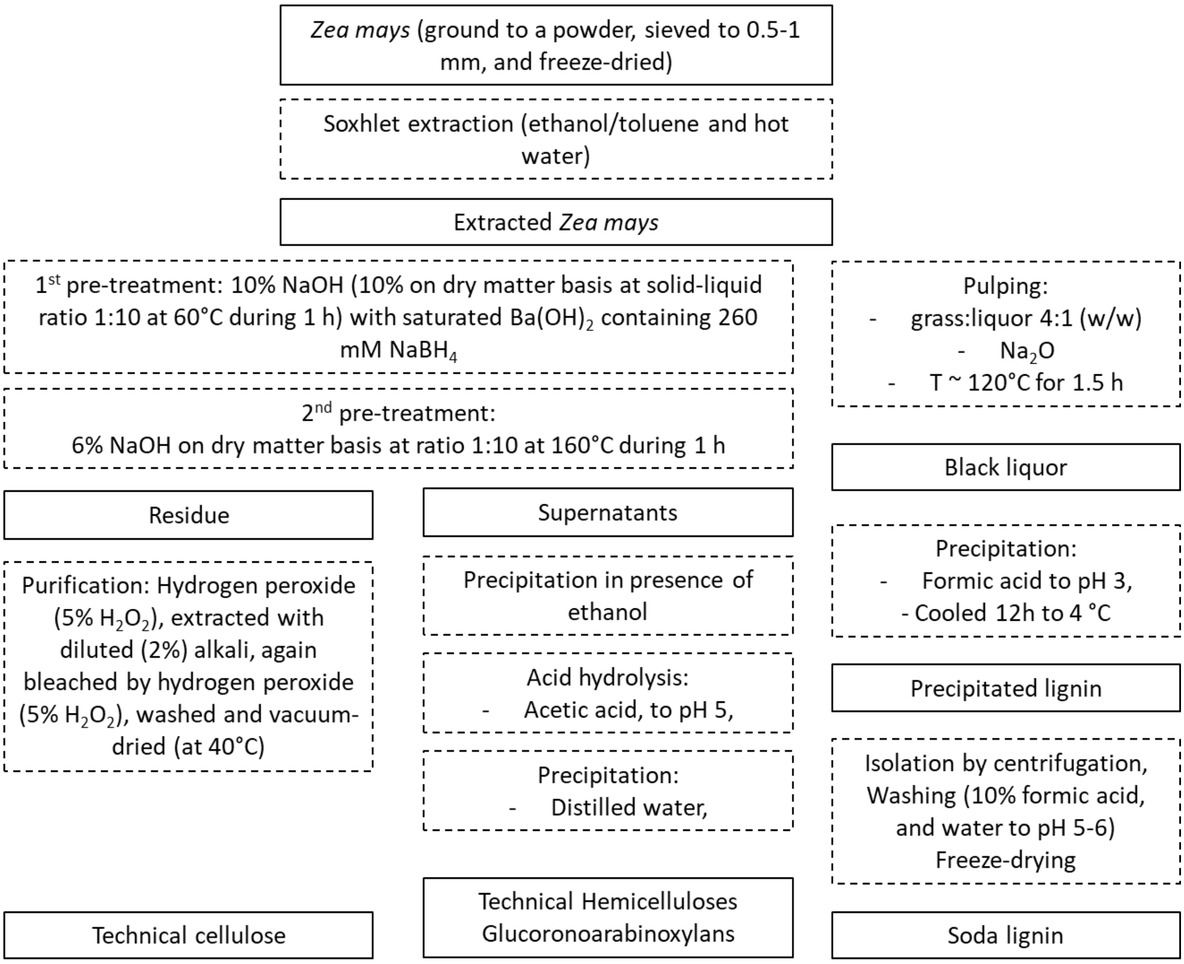

Table S2. Relative distribution between concentrations of major inorganics when detected in samples.

Sample	Si	Na	Mg	P	S	K	Ca
Cellulose	**100.0**	n.d.	n.d.	n.d.	n.d.	n.d.	n.d.
Hemicelluloses	6.4	**32.3**	3.1	21.0	2.5	3.9	**30.9**
Lignin	**60.4**	**39.6**	n.d.	n.d.	n.d.	n.d.	n.d.
Maize	n.d.	n.d.	4.3	12.5	8.2	**41.2**	**33.8**
Cellulose char	90.1	n.d.	n.d.	n.d.	n.d.	n.d.	9.9
Hemicelluloses char	3.9	37.8	3.2	20.8	1.1	3.2	30.0
Lignin char	**49.3**	**50.7**	n.d.	n.d.	n.d.	n.d.	n.d.
Maize char	13.9	0.0	2.5	9.2	5.4	41.2	27.8

n.d.: not detected

Table S3. List of compounds detected and identified by Py-GC/MS

RT (min)	Coumpound name	m/z of detected fragments	Cell	Hemi	Lig	MX
2.06-2.08	2-butanone	72 58 56 44 43 42	√	√	√	√
2.26-2.273	3-methylfuran	82 81 53 39	√	√	√	√
2.48-2.507	2,3-butanedione	86 43	√	√	√	√
3-3.067	acetaldehyde, hydroxy-	60 56 42 31	√	√	√	√
3.414-3.434	acetic acid	62 60 45 43	√	√	√	√
4.04-4.087	1-hydroxy-2-propanone	43 31 42 74	√	√	√	√
4.527-4.534	methyl formate	62 60 44 42 31	√			√
6.308-6.461	1-hydroxy-2-butanone			√	√	√
6.501	acetic anhydride	43 42 15				√
7.388-7.428	5-hydroxymethyl-2(5H)-furanone	84 54 95 96 55 39	√		√	√
8.615-8.795	furfural	96 95 39 38 67 37	√	√	√	√
9.022-9.055	2-propylfuran	81 53 110 51	√			√
10.022-10.102	5-methyl-2(3H)-furanone		√		√	√
10.442-10.756	2-furanmethanol		√		√	√
10.556	2-methyl-2-cyclopenten-1-one	96 67 39 53 41 40 95 68 42		√		
10.822-11.643	acetol acetate	116 100 86 73 43	√	√	√	√
11.316-11.443	2-acetylfuran	95 110 67 39 43	√	√	√	√
11.99-12.069	4-cyclopenten-1,3-dione	96 84 68 57 42 31	√	√		√
12.196	butanoic acid	57 31 41 42 39 43 116			√	
13.51-13.703	2-hydroxy-2-cyclopenten-1-one	98 69 55 43	√	√	√	√
14.35	3-methyl-2-cyclopenten-1-one	96 39 67 81 53 95 40 41 43 42		√		
14.53-14.657	2-furancarboxyaldehyde-5-methyl	110 73 70 58 53 44	√	√	√	√
15.164	2-furanmethanol	48 70 55 44 41 31		√		
15.557-15.691	4-hydroxybutanoic acid		√	√	√	√
16.044-16.257	2(5H)-furanone	55 84 54 39	√	√	√	√
17.524	3-methyl-2,5-furandione	112 83 68 40 39		√		
18.085	2,3-dimethyl-2-cyclopenten-1-one	110 67 95 39 109 41 79 54 53 81		√		
17.685-18.165	tert-butyl-n-butyl-amine	114 58 57 42	√		√	√
18.632	3-methyl-1,2-cyclopentanedione	112 83 55 43 41 69 56 39 42 84		√		
18.985-19.105	2-hydroxy-3-methyl-2-cyclopenten-1-one	112 41 69 83 55 56 84 43 39 97	√		√	√
19.972-20.099	3-ethyl-2-hydroxy-2-cyclopenten-1-one	126 84 83 43 41 55 97 39 70 57		√		√
20.739-21.025	phenol	94 66 39 65		√	√	√
20.899-21.326	2-methoxyphenol	124 109 81 53 43		√	√	√
21.132	4-hydroxy-2,5-dimethyl-3(2H)-furanone	43 128 44 72 57 42	√			
21.199	cyclohexanone,4-methylidene	110 81 67 53 41		√		
23.306-23.426	2-methylphenol	108 90 79 68 63 55 44 39		√	√	√
24.053	2,5-furandicarboxaldehyde	124 123 95 67 125 53 50				√
24.473	4-methyl-5H-furan-2-one	69 41 39 40 98 38 27 68 29 37				√
25.267-25.493	p-cresol	107 108 77 79 39 53 41 85		√	√	√
25.44	3-methylphenol	107 108 90 79 51 39		√		√
25.56	5-hydroxymethyldihydrofuran-2-one	85 29 31 57 86 27 56				√
26.134-26.367	levoglucosenone	98 39 96 68 42 53 97 41 43 85	√			√
26.321-26.427	2,3-dihydroxybenzaldehyde			√		√
26.714	butanal	44 57 43 41 42 39		√		
26.627-26.874	2-methoxy-4-methylphenol (creosol)	123 138 95 67 77 39			√	√
27.761-27.868	2,5-dimethylphenol	122 107 12 77 91 79		√	√	
29.968-30.242	4-ethylphenol	107 122 85 77 39 43		√	√	√
30.398	5-acetyldihydro-2(3H)-furanone	85 29 43 57 27 128 42				√
31.069-31.342	4-ethyl-2-methoxyphenol	137 152 138 122 91			√	√
31.515	heptanal	43 41 69 57 44 70 85 42 39 40	√			
31.655	3-methyl-2,4(3H,5H)-furandione	114 56 42 84 107 86 55		√		
31.869	2H-pyran-3(4H)-one, dihydro-6-methyl	42 56 114 41 59 43 84 55				√
31.982	2,3-dihydro-1H-inden-1-one	132 104 78 63 51 42		√		
32.076-32.149	2,3-anhydro-d-mannosan		√			√
32.322-32.482	3-pyridinol			√		√
33.322-33.496	1,4:3,6 -dianhydro-α,D, glucopyranose	144 114 98 86 69 60 57 41	√			√
34.076-34.59	2-methoxy-4-vinylphenol	150 135 107 77 79 39 151 136 51		√	√	√
34.323	D-erythro-Pentose, 2-deoxy-	116 103 85 73 57 43 31	√			
34.676-34.983	2,3-dihydrobenzofuran	120 91 119 92 39		√	√	√
35.496	eugenol	57 164 73 43 44 45 86 42 55 60			√	
36.91-37.044	2,6-dimethoxyphenol	154 139 93 111 117 96 65 90 107 39		√		√
37.044	5-hydroxymethylfurfural	97 41 126 39 69 38 53 125 51	√			
37.17-37.35	catechol	110 92 81 64 53 39		√		√
37.484	2,6-dimethoxyphenol	154 139 93 96 111 39 65			√	
38.891	2(3H)-furanone,3-acetyldihydro-3-methyl	43 100 99 55		√		
38.958	3,4-dimethoxyphenol	154 139 111 81 65 53			√	
40.705-40.951	4-(1-Propenyl)-2-methoxyphenol (isoeugenol)	165 149 131 103 91 77 121 104 55			√	√
41.598	4-methoxy-3-(methoxymethyl)phenol	68 125 65 139 153 135 110 77 93				√
41.992	sucrose	73 57 31 43 60 61 44 71 86 45				√
42.005	1,2,4-trimethoxybenzene	168 153 125 107 39 79 53 169 65			√	
42.132	vanillin lactoside	151 152 123 81 109			√	
43.025	4-O-methylmannose	87 74 71 60 45 43 73 88 57 41			√	√
42.992	ethanone, 1-(2,4,6-trihydroxyphenyl)-	168 153 138 123 107 96			√	
42.012	sucrose	86 73 60 57 43 31	√			
43.199-43.219	3-hydroxybenzaldehyde	122 121 93 65 39		√		√
44.193	1,6:2,3-dianhydro-4-deoxy-β-hexopyranose	81 71 54 39	√			
44.94-44.966	2-methyl-1,4-benzenediol	124 107 95 77 67 51		√		√

RT (min)	Coumpound name	m/z of detected fragments	Cell	Hemi	Lig	MX
45.133-45.453	1,2,3-trimethoxy-5-methylbenzene	182 167 151 138 123 107 91			√	√
45.579	2'-Hydroxy-6'-methoxyacetophenone	15 166 136 108 43 152 65				√
47.207	3-hydroxy-4-methylbenzaldehyde	136 107 89 77 63		√		
47.78-48.12	2,4-dimethoxyacetophenone	180 165 137 122 105 91 77 65 51		√	√	√
48.434	ethyl-homovanillate	137 131 122 106 94			√	
48.767	2-methyl-3,4-dithiaoctane	164 122 57 43	√			
48.694	3,4-Dihydro-6-hydroxy-2H-1-benzopyran-2-one	164		√		
48.774-50.194	4-allyl-2,6-dimethoxyphenol	60 194 62 57 73 32 44 45 91 76		√	√	√
51.908-53.881	1,6-Anhydro-β-D-glucopyranose (LVG)		√			√
54.822	4-hydroxy-3,5-dimethoxybenzaldehyde	182 181 65 167 93 39 183 53 111 51				√
57.843-58.277	1-(4-hydroxy-3,5-dimethoxyphenyl)ethanone	182 181 167 81 196			√	√
59.7-59.944	desaspidinol	210 167 168 211 195			√	√
	3-methyl-1-(2,4,6-trihydroxy-3-methylphenyl)-1-butanone	167 210 168 123 43			√	
	n-hexadecanoic	43 41 73 60 57 55 129 256 71 69			√	
66.832	9,12-octadecadenoic acid	67 81 82 95 68			√	

Supplementary Information: Reconstruction of mass spectrum

Mass spectrometry spectra are used to track and assess the inter-exchange of carbon atoms between organic compounds evolved from fast pyrolysis of biomass. To conduct both fast pyrolysis of biomass and volatiles analysis, the Pyroprobe was used (Figure S2). The biomass is heated in a controlled manner (1), volatiles released entered in the trapping zone (2) where they are adsorbed on a Tenax® pre-column, desorbed and injected in the GC system (3) to be separated before characterization. Volatiles are electronically ionized. Ions are subsequently filtered and selected according to their mass-to-charge ratio (4). Their respective abundance that results from a manipulation of ions into electrical signals is displayed as a series of ion peaks and made up a mass spectrum (5). All ion peaks in each spectrum are summed resulting in a total ion current and subsequently in a complete mass spectrum. When this total ion current is plotted against the time, a chromatogram (e.g. total ion current) is displayed.

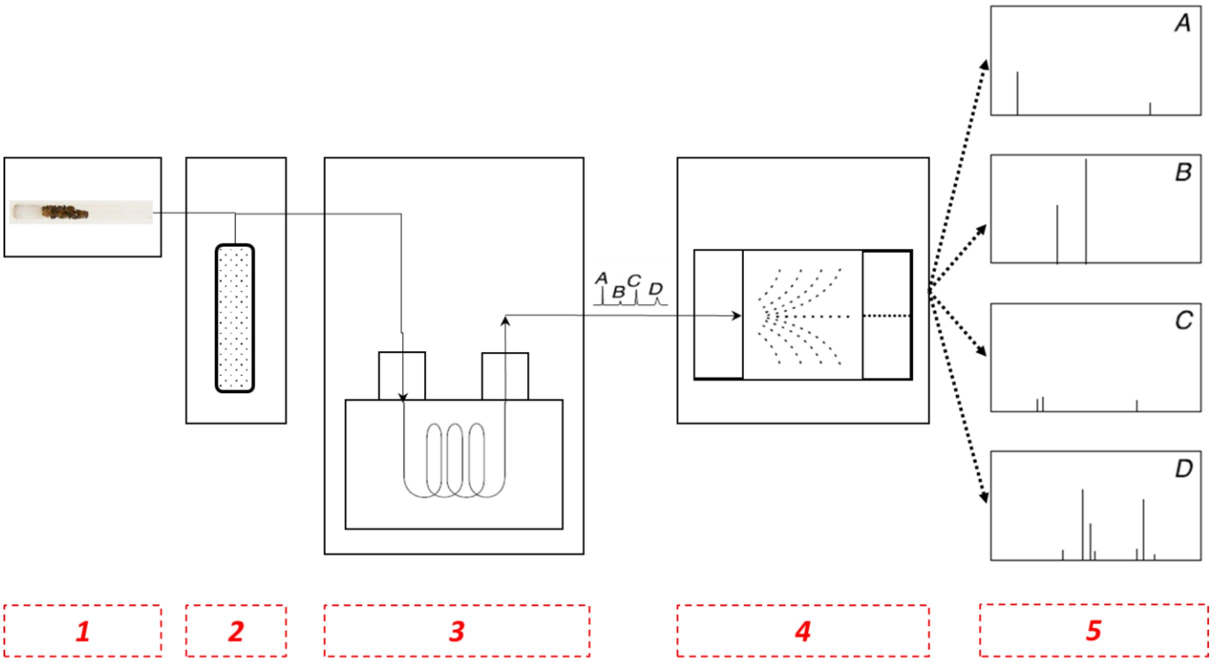

Figure S2. Schema of the pyroproprobe showing the different sections: (1) quartz tube reactor inserted in the oven; (2) trap; (3) gas chromatogram; (4) mass spectrometer (MS) and (5) resulting single MS spectrum.

Mass-to-charge ratio (m/z) and absolute intensity of ionic fragments corresponding to the fragmentation of Furfural were plotted. The intensities were normalized and any m/z intensity ratio negligible (less than 0.02) were set to zero. The distribution of ion peaks depend on the chemical formula and isotopic composition of the chemicals. The isotopic natural abundance percentage for Carbon-12 being of 98.90% and 1.10% for Carbon-13, furfural produced from the pyrolysis of unlabelled biopolymers will display ions fragments ranging from 29 to 97 with m/z = 96 and 97 the two more intense peaks (Figure S3a). On the other hand, the conversion of Carbon-13 material enriched at a high level of 98% leads to a different fragmentation pattern, displaying in general heavier fragments such as 100 and 101 (Figure S3b). It is therefore not surprising that MS spectra of Furfural resulting from the conversion of mixtures (e.g. Cell-13C+Lig-12C, Cell-12C+Lig-13C) give peaks in the m/z range of their respective unlabelled and Carbon-13 enriched components (Figure S3c-d).

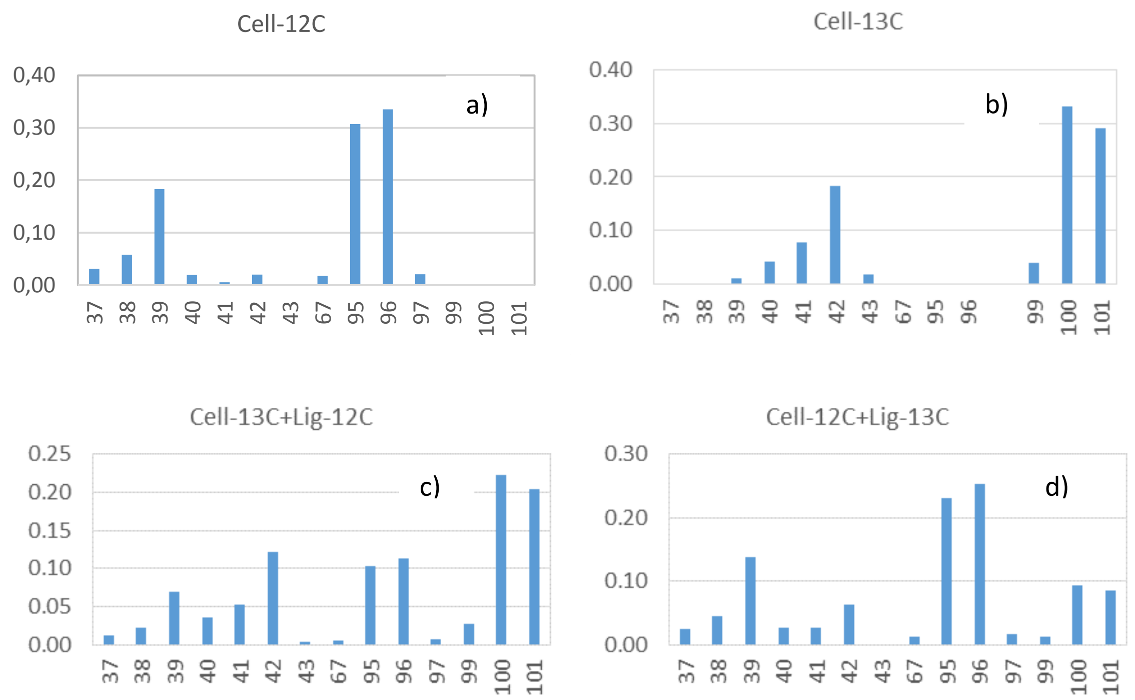

Figure S3. Normalized intensities for m/z ratio of furfural.

In order to reconstruct the corresponding fragmentation patterns for Furfural obtained from those mixtures based on the hypothesis that no chemical interactions occurred between hot volatiles, the 'predicted' and absolute m/z intensities were calculated multiplying the experimental intensity at m/z ratio of 96 for unlabelled polymers and 101 for those enriched by the ratio of relative intensities 96/95 or 101/100. The same method was used to determine intensities corresponding to the nominal mass of M-1 by considering the ratio of relative intensities between the nominal mass M and M-1. Both values are summarized in Table S4 and normalized intensities shown in Table S5.

Table S4. Experimental and calculated intensities to construct mass spectrometer spectra.

	Experimental													Calculated	
m / z	Cell-^{12}C			Cell-^{13}C			Lig-^{12}C			Lig-^{13}C				Cell-^{13}C+Lig-^{12}C	Cell-^{12}C+Lig-^{13}C
	Absolute intensity	Relative intensity	Ratio	Absolute intensity	Relative intensity	Ratio	Absolute intensity	Relative intensity	Ratio	Absolute intensity	Relative intensity	Ratio		Absolute intensity	Relative intensiy
**37**	13400000	0.0342	0.5390				13280000	0.0373	0.5529					922564	3905342
**38**	24870000	0.0635	0.3171				24020000	0.0675	0.3285					1668673	7063729
**39**	78420000	0.2002	0.5968				73110000	0.2053	0.6154					5078963	21499968
**40**				16500000	0.0448	0.6				4750000	0.0364	0.6124		8758235	1393207
**41**				29740000	0.0809	0.4				7757000	0.0594	0.4653		15814809	2275180
**42**				69350000	0.1887	4.5				16670000	0.1277	2.7128		36878177	4889422
**95**	131400000	0.3355	0.9150				118800000	0.3336	0.9362					30640851	36025195
**96**	143600000	0.3666					126900000	0.3564						32730000	39370000
**99**				15520000	0.0422	0.1				6145000	0.0471	0.1222		8253054	1802369
**100**				125700000	0.3420	1.1				50280000	0.3851	1.1181		66843357	14747458
**101**				111000000	0.3014					45000000	0.3444			58920000	13190000
Total	391700000			367580000	1		356110000	1		130572000	1				
Calculated total														266508682	146161870
Actual total														283996000	155089000
Error (%)														-6.157592923	-5.756133547

Table S5. Mass-to-charge ratio (m / z) and corresponding normalized intensity for furfural produced from the fast pyrolysis of mixtures.

	Calculated		Experimental	
m / z	Cell-^{13}C + Lig-^{12}C	Cell-^{12}C + Lig-^{13}C	Cell-^{13}C + Lig-^{12}C	Cell-^{12}C + Lig-^{13}C
37	0	3	1	2
38	1	5	2	4
39	2	15	7	14
40	3	1	3	3
41	6	2	5	3
42	14	3	12	6
95	11	25	10	23
96	12	27	11	25
99	3	1	3	1
100	25	10	22	9
101	22	9	20	8
	100	100	98	100

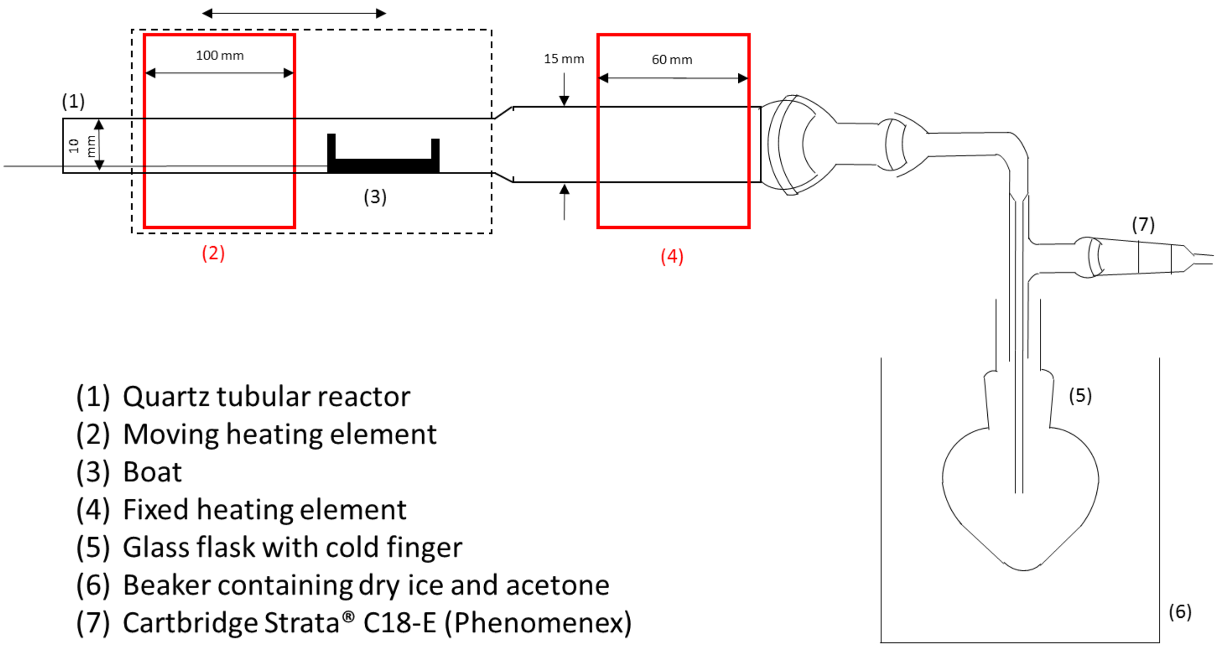

Figure S4. Description of the tubular microreactor.

Table S6. List of compounds detected and identified by GC/MS

RT (min)	Compound name	Cell	Hemi	Lignin	MX	Calibration
5.488	poss: 2-Butenone (NIST MQ 78)	√	√	√	√	
5.554	2,3-butandione	√	√	√	√	c
5.585	2-butanone	√	√	√	√	c
5.750	poss: Glycerin	√		√	√	
5.945	3-pentanone	√		√	√	
6.104	benzene (NIST MQ 97)	√	√	√	√	
6.554	acetaldehyde, hydroxy-	√	√	√	√	c
6.732	2-methylbutanal (NIST MQ 82)			√		
6.876	2,5-dimethylfuran (NIST MQ 97)			√		
7.358	Cis-crotonaldehyde	√	√		√	
7.612	Acetic acid	√	√	√	√	c
8.308	poss: 2,3-pentanedione (or methyl-isobutyl ketone) (NIST MQ 90)	√	√	√	√	
8.321	1-octene			√		
8.995	Acetol (Hydroxypropanone)	√	√	√	√	c
9.084	poss: cyclotrisiloxane, hexamethyl- (NIST MQ 88)	√	√	√	√	
9.347	toluene		√	√	√	c
10.183	1,2-dihydroxyethylene,	√		√	√	
10.565	Acetoin (Butanone-2, 3-hydroxy-)	√	√	√	√	c
10.577	poss: Propanoic acid, 2-oxo-, methyl ester (NIST MQ 74)			√		
11.517	propanoic acid	√	√	√	√	c
13.119	cyclopentanone			√	√	c
13.347	1-hydroxy-2-butanone	√		√	√	c
13.483	Ethylbenzene			√		c
13.580	3-hydroxypropionaldehyde	√		√	√	
13.942	p-xylene		√	√		c
13.936	m-xylene			√		c
14.104	2(3H)-furanone	√	√		√	
14.811	3(2H)-furanone	√	√			c
14.829	butanoic acid, 2-propenyl ester			√		
15.108	3-furaldehyde	√	√		√	c
15.157	o-xylene (NIST MQ 94)			√		
15.733	poss: oxopropanoic acid methylester, 2- (NIST MQ 77)	√	√	√	√	
15.765	butanedial or propanal (NIST MQ 88)	√	√	√	√	
15.762	styrene			√		
15.824	cyclotetrasiloxane, octamethyl- (NIST MQ 89)			√		
16.314	2-cyclopenten-1-one	√	√	√	√	c
16.484	2-furaldehyde	√	√	√	√	c
17.241	2-propylfuran,	√				
18.660	2-furfuryl alcohol	√	√	√	√	c
19.091	1-Acetoxy-2-propanone	√	√	√	√	c
19.207	2-methyl-2-cyclopentene-1-one	√	√	√	√	c
19.508	poss: aliphatic ketone (unknown unspecific spectrum)	√	√	√	√	
20.041	1-(2-furanyl)-ethanone, (Acethylfuran)	√	√	√	√	c
20.556	poss: 5H-Furan-2-one, 4-methyl- (NIST MQ 74)	√		√	√	
20.900	4-Cyclopentene-1,3-dione (NIST MQ 86)	√	√	√	√	
21.669	poss: 2(3H)-Furanone, dihydro-4-hydroxy- (NIST MQ 82)	√		√	√	
22.001	2-hydroxy-2-cyclopenten-1-one	√	√	√	√	
23.010	Furan-x-one, x,x-dihydro-x-methyl-	√	√	√	√	
23.018	1-methoxy-3-methylbenzene			√		c
23.451	Anhydrosugar unknown (unspecific spectrum)	√		√		
23.475	5-methyl-2-furaldehyde	√	√		√	c
23.720	poss. propanoic acid, ethenyl ester (NIST MQ 75)	√		√		
23.847	poss: 1-(acetyloxy)- butan-2-one (NIST MQ 77)	√		√		
23.856	Isomere of 2,3-dimethyl-2-cyclopenten-1-one		√	√	√	c
24.170	3-methyl-2-cyclopenten-1-one		√			c
24.477	Butyrolactone, gamma-	√	√	√	√	c
24.906	2(5H)-furanone,	√	√	√	√	c
25.680	poss: 5-Methyl-2(5H)-furanone (NIST MQ 84)	√	√	√		
26.006	5,6-dihydro-3-hydroxy-(4H)-pyran-4-one	√	√	√	√	
26.200	3-methyl-2,5-furandione (NIST MQ 67)	√	√	√	√	
26.197	2-hydroxy-benzaldehyde (Salicylaldehyde)			√		
26.851	2,3-dimethyl-2-cyclopenten-1-one		√	√		c
26.872	2-hydroxy-3-methyl-2-cyclopenten-1-one	√	√	√	√	c
27.029	poss: unknown cyclic compound derived from sugars	√				
27.130	3-methyl-2(5H)-furan-2-one	√	√	√	√	c
27.139	benzeneacetaldehyde (NIST MQ 82)			√		
27.248	acetophenone			√		c
27.773	2,5-dihydro-3,5-dimethyl-furan-2-one		√			
28.366	poss: 2-cyclohexene-1,4-dione (NIST MQ 82)	√				
28.879	phenol	√	√	√	√	c
29.367	similar to: 4-hydroxy-2,5-dimethyl-3(2H)-furanone (NIST MQ 78)	√			√	
29.449	Guaiacol		√	√	√	c
29.881	3-ethyl-2-cyclopenten-1-one		√			
30.805	poss: 3-furancarboxylic acid, methyl ester (NIST MQ 84)	√				
31.192	o-cresol		√	√	√	c
31.186	2-hydroxy-gamma-butyrolactone		√	√		c
31.669	2,6-dimethyl-phenol			√		

RT (min)	Compound name	Cell	Hemi	Lignin	MX	Calibration
32.169	2,5-furandicarboxaldehyde (NIST MQ 82)	√				
32.581	4-methyl-(5H)-furan-2-one (NIST MQ 88)	√			√	
32.925	p-cresol		√	√	√	c
33.008	m-cresol		√	√	√	c
33.214	Lactone derivative (unspecific spectrum)	√		√	√	
33.205	4-methyl-guaiacol			√	√	c
33.731	poss: 4H-Pyran-4-one, 2,3-dihydro-3,5-dihydroxy-6-methyl- (NIST MQ 68)	√				
34.546	Anhydrosugar unknown (unspecific spectrum)	√	√	√	√	
34.537	2-ethyl-phenol			√		c
34.724	poss: Succinic anhydride = 2,5-Furandione, dihydro- (NIST MQ 88)			√		
35.070	2,4-dimethylphenol		√	√		c
35.127	2,5-dimethylphenol		√	√		c
35.122	1,2-dimethoxy-3-methyl-benzene (NIST MQ 94)			√		
35.425	2,4,6-trimethylphenol			√		c
35.958	1,3-dimethoxy-5-methyl-benzene, (NIST MQ 92)			√		
36.946	4-ethylphenol		√		√	c
36.926	3-ethylphenol			√		c
37.004	4-ethyl guaiacol			√		c
38.066	3-pyridinol			√		
38.726	poss: 2,3-anhydro-d-galactosan (NIST MQ 78)	√		√	√	
38.956	poss: 2,3-anhydro-d-mannosan (NIST MQ 84)	√			√	
39.452	2,3-dihydro-1H-inden-1-one,	√				c
39.734	1,4:3,6-dianhydro-alpha-D-glucopyranose	√		√	√	
40.002	1,5-anhydro-beta-D-arabinofuranose	√	√	√	√	
40.653	4-vinylguaiacol		√	√	√	
40.819	4-vinylphenol		√	√	√	
42.366	5-(hydroxymethyl)-2-furaldehyde	√		√	√	c
42.394	Cis-4-propenylphenol			√		
42.659	syringol			√	√	c
42.904	Lactone derivative = Furanone derivative (unspecific spectrum)		√	√	√	
44.714	3-methoxy-5-methylphenol, (NIST MQ 88)			√	√	
45.140	5-hydroxy-2-(hydroxymethyl)-2,3-dihydro-4H-pyran-4-one	√			√	
46.332	1,5-anhydro-beta-D-xylofuranose	√	√	√	√	
46.224	4-methyl-1H-indole (NIST MQ 82)			√		
46.325	4-methyl-2,6-dimethoxyphenol			√		c
46.975	vanillin			√		c
46.983	hydroquinone			√		
47.519	poss: Guaiacol, dimethyl- (NIST MQ >80)			√		
47.538	hydroquinone		√			
48.002	4-hydroxybenzaldehyde		√			
48.045	benzenediol, methyl-			√		
49.140	4-ethyl-2,6-dimethoxyphenol			√		c
49.481	Anhydrosugar unknown (unspecific spectrum)			√		
49.642	phenylethanone, 4-hydroxy-3-methoxy (Acetoguaiacone)			√		
50.505	anhydrosugar unknown (unspecific spectrum)			√		
50.910	4-vinylsyringol			√		
51.775	Guaiacyl acetone			√		c
52.139	4-allyl-syringol			√		c
52.429	3,4-dihydro-6-hydroxy-2H-1-benzopyran-2-one (NIST MQ 83)			√		
52.870	Syringol, 4-(1-propenyl)-, cis			√		
54.215	Anhydrosugar unknown (unspecific spectrum)	√				
54.621	Anhydrosugar unknown (unspecific spectrum)	√				
55.834	Syringol, 4-(1-propenyl)- trans			√		
56.045	1,6-anhydro-β-D-glucopyranose (Levoglucosan)	√			√	c
56.652	Acetosyringone			√		c
59.913	Syringyl acetone			√		
59.915	Anhydrosugar unknown (unspecific spectrum)	√				
61.368	1,6-anhydro-α-D-galactofuranose	√			√	
68.216	Fluoranthene	√	√	√	√	
69.398	Naphthalene, 1-phenyl- (impurity in IS = Fluoranthene)	√	√	√	√	

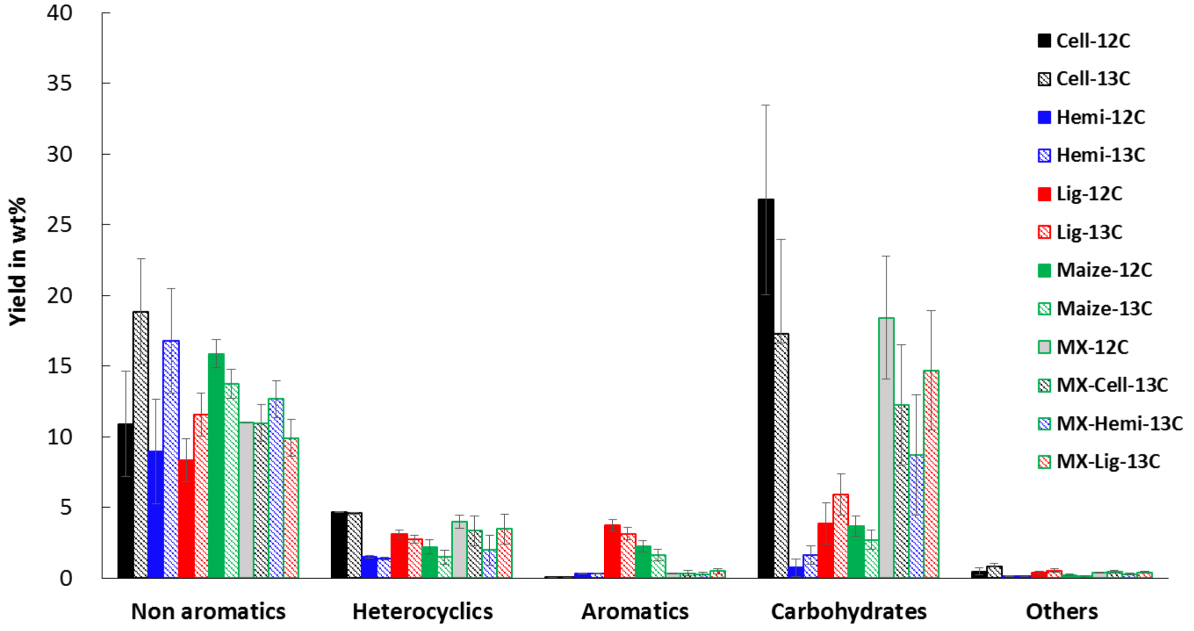

Figure S5. Yields of organic compounds from the fast pyrolysis of control and Carbon-13 enriched materials classified by chemical families.

Figure S6. Quantitative ^{13}C-NMR spectrum for fast pyrolysis condensates produced by pyrolysis of Carbon-13 enriched *Zea Mays* at 550 °C.

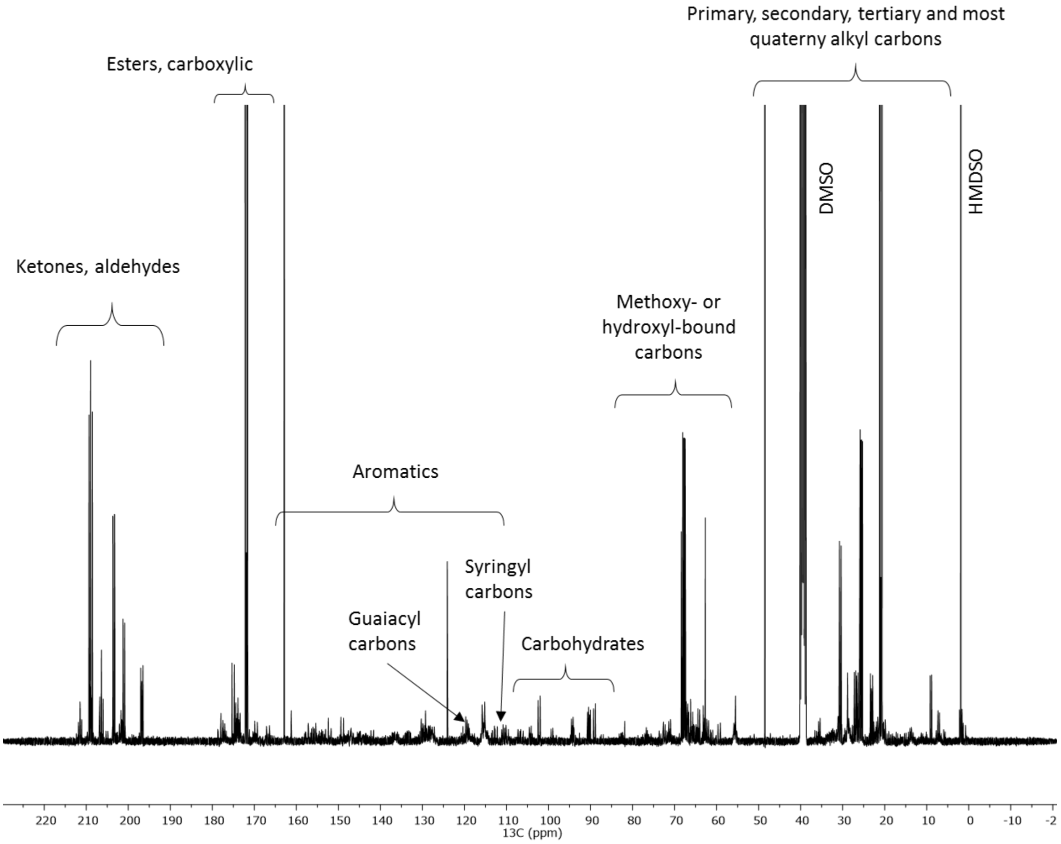

Supplementary Information: Determination of the Carbon source distribution based on Quantitative based on liquid-state ^{13}C NMR analysis.

After collecting ^{13}C-NMR spectra and integrating the regions, the quantification of functional groups in bio-oil was possible. In addition to provide an unbiased amount of structural details, the size of peaks can be correlated to the amount of carbon atoms when the ^{13}C-NMR spectra are recorded under quantitative conditions. Two different internal standards were used, Hexamethyldisiloxane (HMDSO) for liquids issued from Carbon-13 materials and Demethyl sulfoxide (DMSO) for liquids produced from unlabelled materials due to the peak intensity.

Quantitative estimates of the various carbon-containing functional groups were made by performing the following calculations.

The signal area of the selected internal standard signal, which was 0.709541078 g of DMSO (ca. 0.000200631 of ^{13}C mol per unit area) or 0.002862703 g of HMDSO (ca. 0.0000013 of ^{13}C mol per unit area), was integrated and calibrated to 1.0.

Because those molecules, DMSO and HMDSO, contain respectively 2 and 6 carbons and have a molecular weight of 84.17 g/mol and 162.38 g/mol, 0.709541078/84.17*2 = 0.01686 moles or 0.002862703/162.38*6 = 0.000106 moles was the numbers of carbon moles from the internal standard present in the prepared sample and when multiplied by the natural abundance of Carbon 13 (1.19/100), corresponded to 0.000200631 of ^{13}C mol in the case of DMSO and $1.25876*10^{-6}$ of ^{13}C mol for HMDSO.

For each spectrum, this factor, mole of ^{13}C per area unit, was multiplied by the integration region of interest and divided by the weight of condensates produced; thus leading to the number of moles by functional group per gram of sample.

The quantitative results from all ^{13}C-NMR experiments are shown in Table S7.

Table S7. Results from quantitative ^{13}C-NMR analysis of fast pyrolysis bio-oils derived from unlabelled technical biopolymers (Cell-^{12}C, Hemi-^{12}C and Lig-^{12}C) and Carnon-13 enriched biopolymers (Cell-^{13}C, Hemi-^{13}C and Lig-^{13}C).

Integration regions	Cell-^{12}C	Cell-^{13}C	Hemi-^{12}C	Hemi-^{13}C	Lig-^{12}C	Lig-^{13}C	MX-^{12}C	MX-Cell-^{13}C	MX-Hemi-^{13}C	MX-Lig-^{13}C	Maize-^{13}C	Maize-^{12}C
Internal standard	1	1	1	1	1	1	1	1	1	1	1	1
Ketones, Aldehydes	5.235	0.070	1.768	0.027	1.003	0.035	1.724	8.720	2.809	0.337	4.842	10.537
Ester, carboxylic acids	1.028	0.030	0.638	0.020	0.722	0.016	1.097	3.788	2.636	0.012	7.283	27.474
total aromatic carbon	2.973	0.052	3.862	0.042	0.847	0.149	4.539	9.237	4.377	2.218	13.124	23.959
Carbohydrate-type carbon	0.515	0.113	5.292	0.004	2.499	0.015	5.320	11.660	0.107	0.312	2.055	28.613
Methoxy- or hydoxy carb.	38.667	0.335	2.170	0.053	2.357	0.085	2.220	58.769	6.837	3.276	8.251	11.611
Alkyl carbons I - alkanes	1.030	0.006	0.488	0.001	0.920	0.002	4.619	4.488	1.459	0.395	0.464	50.419
Alkyl carbons II - alkanes	6.005	0.096	0.133	0.069	3.109	0.363	3.134	16.686	8.878	7.262	20.826	32.525

Results (mol ^{13}C/g condensates)	Cell-^{12}C	Cell-^{13}C	Hemi-^{12}C	Hemi-^{13}C	Lig-^{12}C	Lig-^{13}C	MX-^{12}C	MX-Cell-^{13}C	MX-Hemi-^{13}C	MX-Lig-^{13}C	Maize-^{13}C	Maize-^{12}C
Correction factor-HMDSO (mol/unit area)	0.000018	0.000018	0.000018	0.000018	0.000018	0.000018	0.000018	0.000141	0.000140	0.000141	0.000141	0.000018
Correction factor-HMDSO (^{13}Cmol/unit area)	0.000001	0.000001	0.000001	0.000001	0.000001	0.000001	0.000001	0.000010	0.000010	0.000010	0.000010	0.000001
Correction factor-DMSO (mol/unit area)	0.008430	0.008430	0.008430	0.008430	0.008430	0.008430	0.008430	0.008059	0.008200	0.008059	0.008059	0.008430
Correction factor-DMSO (^{13}Cmol/unit area)	0.000201	0.000201	0.000201	0.000201	0.000201	0.000201	0.000201	0.000192	0.000195	0.000192	0.000192	0.000201
Ketones, Aldehydes	0.0005	0.0010	0.0002	0.0005	0.0001	0.0005	0.0002	0.0059	0.0019	0.0002	0.0033	0.0009
Ester, carboxylic acids	0.0001	0.0004	0.0001	0.0003	0.0001	0.0003	0.0001	0.0026	0.0018	0.0000	0.0049	0.0025
total aromatic carbon	0.0003	0.0008	0.0004	0.0007	0.0001	0.0023	0.0005	0.0062	0.0030	0.0015	0.0088	0.0022
Carbohydrate-type carbon	0.0000	0.0017	0.0006	0.0001	0.0003	0.0002	0.0006	0.0079	0.0001	0.0002	0.0014	0.0026
Methoxy- or hydoxy carb.	0.0035	0.0050	0.0003	0.0009	0.0003	0.0013	0.0003	0.0397	0.0047	0.0022	0.0055	0.0010
Alkyl carbons I - alkanes	0.0001	0.0001	0.0001	0.0000	0.0001	0.0000	0.0006	0.0030	0.0010	0.0003	0.0003	0.0045
Alkyl carbons II - alkanes	0.0005	0.0014	0.0000	0.0012	0.0004	0.0057	0.0004	0.0113	0.0061	0.0048	0.0140	0.0029
Total	0.0051	0.0105	0.0017	0.0038	0.0013	0.0104	0.0027	0.0766	0.0187	0.0092	0.0382	0.0166

Carbon distribution (%)	Cell-^{13}C	Hemi-^{13}C	Lig-^{13}C
Ketones, Aldehydes	50.9	22.5	26.6
Ester, carboxylic acids	42.2	33.2	24.6
Total aromatic carbons	20.3	19.1	60.6
Carbohydrate-type carbons	84.6	3.8	11.6
Methoxy- or hydoxy carbons	69.1	12.6	18.3
Alkyl carbons I - alkanes	61.7	14.1	24.2
Alkyl carbons II - alkanes	17.3	14.4	68.3

Table S8. Percentage of match between quantitative ^{13}C-NMR results; Comparison between the chemical analysis of fast pyrolysis bio-oils produced from technical biopolymers and their mixture, and the bio-oil generated from the Carbon-13 enriched maize (Maize-^{13}C).

Functional group	Cell-^{13}C	Hemi-^{13}C	Lig-^{13}C	MX-^{13}C	Maize-^{13}C
Ketones, Aldehydes	3.0	40.6	-42.7	-33.6	0.0
Ester, carboxylic acids	-63.8	-17.7	-66.7	-52.7	0.0
Total aromatic carbons	-57.6	-21.2	-15.0	-51.2	0.0
Carbohydrate-type carbons	423.7	27.8	8.2	139.6	0.0
Methoxy- or hydoxy carbons	187.9	49.1	-2.2	126.7	0.0
Alkyl carbons I - alkanes	-91.4	56.9	-29.8	1.4	0.0
Alkyl carbons II - alkanes	-61.0	-22.4	59.4	-8.6	0.0
